# A GT 3D Bounding-Box-Based Framework for Geometric Quantification and Spatial Validation of Vehicle Frontal Blind Spots

**DOI:** 10.3390/s26217093

**Published:** 2026-11-09

**Authors:** Heeju Je, Seoyoung Choi, Beom Kwon

**Affiliations:** 1 Division of Global Regional Studies, Dongduk Women’s University, Seoul 02748, Republic of Korea; 20210154@dongduk.ac.kr; 2 Division of Information Science, Dongduk Women’s University, Seoul 02748, Republic of Korea; 20220856@dongduk.ac.kr; 3 Division of Interdisciplinary Studies in Cultural Intelligence, Dongduk Women’s University, Seoul 02748, Republic of Korea

**Keywords:** 3D bounding box, blind spot, roadside perception, spatial validation, vehicle visibility

## Abstract

Vehicle-induced frontal blind spots can restrict a driver’s direct visibility of pedestrians and other vulnerable road users, and their spatial extent depends on vehicle geometry and driver-viewpoint conditions. Conventional approaches based on category-level representative dimensions may not preserve geometric variation among individual vehicles, whereas detailed visibility models typically require vehicle-specific computer-aided design models and driver accommodation information. This study proposes a geometric framework that uses ground-truth (GT) three-dimensional (3D) bounding boxes from the TUMTraf roadside perception dataset as an intermediate representation for frontal blind-spot quantification and spatial placement. Vehicle-specific GT width is used to estimate the blind-spot ground area and volume, while bounding-box position, dimensions, and yaw are used to spatially anchor the resulting geometry. A mean-width baseline comparison, sensitivity analyses of representative visibility parameters, and calibration-based camera projection were conducted to evaluate complementary aspects of the framework. Replacing vehicle-specific GT widths with the mean-width baseline resulted in a mean absolute percentage error of 5.8% and a maximum relative error of 38.3%. Increasing the assumed frontal obstruction height from 1.030 to 1.160 m increased the estimated blind-spot distance from 8.583 to 21.091 m, demonstrating substantial sensitivity to visibility assumptions. In an exploratory spatial validation of 27 vehicles, all three assessors classified 24 cases (88.9%) as Valid or Partially Valid, although agreement on individual four-category verdicts was moderate (Fleiss’ κ=0.512). These qualitative results describe the evaluated sample and do not establish population-level performance or physical blind-spot accuracy. The findings support the feasibility of using GT 3D bounding boxes as a compact geometric representation for vehicle-level blind-spot analysis while highlighting the need for vehicle-specific visibility parameters and independent physical validation.

## 1. Introduction

Vehicle-induced blind spots are an important factor affecting road safety because they can obstruct a driver’s direct view of pedestrians and other vulnerable road users in the vicinity of a vehicle [1]. The extent of these blind spots is closely related to vehicle geometry and structural characteristics, which determine the portion of the surrounding environment that is occluded from the driver’s viewpoint [2]. Therefore, quantitatively characterizing frontal blind spots in terms of distance, ground area, and spatial volume can provide a useful basis for evaluating differences in visibility among vehicles. Such characterization should ideally account for the geometric properties of individual vehicles rather than assuming that all vehicles within the same category have identical dimensions.

Previous studies have investigated vehicle blind spots using geometric or volumetric models [3,4]. One practical approach for road-infrastructure applications is to detect vehicles in two-dimensional (2D) road images, classify their vehicle types, and combine the detected information with representative vehicle dimensions to estimate the corresponding blind-spot regions. This approach enables blind-spot analysis from conventional roadside imagery without requiring detailed three-dimensional (3D) vehicle models. However, representing all vehicles within a category using a single set of representative dimensions inevitably discards vehicle-specific geometric variations. Previous volumetric visibility studies have also demonstrated that differences in vehicle size and structure can lead to substantial differences in the extent of occluded regions [2]. These observations suggest that vehicle-specific geometric information should be considered when blind spots are quantified.

A further limitation of 2D image-based approaches is that a 2D bounding box does not directly provide the physical position, dimensions, or orientation of a vehicle in 3D space. In particular, depth cannot be directly recovered from a monocular image without additional estimation, and reconstructing 3D object information from 2D observations inevitably introduces uncertainty [5]. When such estimated spatial information is subsequently used for geometric blind-spot calculations, errors in vehicle localization, dimensions, or orientation may propagate to the resulting spatial representation. In contrast, 3D traffic datasets acquired using calibrated roadside sensors can provide ground-truth (GT) 3D bounding boxes containing vehicle-specific dimensions, positions, and orientations [6]. These data offer an opportunity to investigate blind-spot geometry while separating the geometric modeling problem from uncertainties associated with 3D object estimation.

Despite extensive research on 3D vehicle perception and visibility analysis, the use of vehicle-level GT 3D bounding-box information for geometric quantification and spatial placement of frontal blind spots has received comparatively limited attention. Existing 3D object detection datasets are primarily used to evaluate perception performance, whereas detailed visibility studies often rely on vehicle computer-aided design models or driver accommodation information that is not available in standard traffic datasets. This leaves a methodological gap between coarse category-level vehicle representations derived from 2D imagery and detailed vehicle-specific visibility models requiring substantially richer geometric information.

To address this gap, this study develops a geometric framework for quantifying and spatially representing vehicle frontal blind spots using GT 3D bounding boxes provided by the TUMTraf dataset. The framework combines vehicle-specific 3D information—including the bounding-box center, length, width, height, and yaw—with literature-based representative parameters describing the driver’s eye position and frontal vision-obstruction point. The resulting model estimates the frontal blind-spot distance, ground area, and volume and places the estimated region in the road coordinate system according to the position and orientation of each vehicle. The use of GT 3D information allows the proposed framework to focus on the geometric feasibility and validity of blind-spot quantification without introducing additional uncertainty from 3D object estimation. To maintain consistent assumptions regarding driver position and frontal vehicle geometry, the present analysis is restricted to vehicles labeled as the CAR class.

The main contributions of this study are summarized as follows:

- GT 3D bounding-box-based blind-spot quantification and spatial representation: A geometric framework is developed that combines literature-based driver-visibility parameters with vehicle-specific GT 3D bounding-box information. Vehicle width is incorporated into the quantitative estimation of blind-spot area and volume, while the bounding-box center, length, height, and yaw are used to determine the spatial reference and placement of the estimated blind-spot region in the road coordinate system.
- Quantitative assessment of information loss caused by representative vehicle dimensions: The blind-spot estimates obtained using individual GT vehicle widths are compared with those obtained using a single mean width for all CAR instances. This controlled comparison quantifies how replacing vehicle-specific width information with a representative value affects the estimated blind-spot area and volume.
- Validity assessment through parameter sensitivity and camera-based spatial projection: The sensitivity of the estimated blind-spot geometry to representative visibility parameters is examined through both frontal-obstruction-height-only and joint sensitivity analyses of driver eye height, eye-to-front distance, and frontal obstruction height. In addition, the GT 3D bounding boxes and estimated blind-spot regions are projected onto calibrated camera images to qualitatively assess their spatial consistency with vehicle positions and orientations. A multi-assessor evaluation is further conducted to examine the reproducibility of these qualitative judgments.

The remainder of this paper is organized as follows. Section 2 reviews previous studies on driver eye position and vehicle visibility, geometric and volumetric blind-spot analysis, and vehicle geometry derived from image and 3D data. Section 3 describes the TUMTraf dataset and preprocessing procedures, the proposed blind-spot geometry, spatial placement using GT 3D bounding boxes, camera projection based on sensor calibration, and the experimental validation procedures. Section 4 presents the overall blind-spot characteristics, sensitivity analysis, mean-width baseline comparison, and qualitative spatial validation results. Section 5 discusses the implications of vehicle-specific geometric information, the sensitivity to visibility assumptions, the interpretation of the spatial validation results, and the limitations and future directions of the proposed framework. Finally, Section 6 concludes the paper.

## 2. Related Work

### 2.1. Driver Eye Position and Vehicle Visibility

The driver’s eye position is a fundamental parameter in the geometric assessment of direct visibility and vehicle-induced blind spots. SAE J941, Motor Vehicle Drivers’ Eye Locations, provides one of the most widely adopted frameworks for representing driver eye locations [7]. Rather than defining the driver’s eye as a single fixed point, SAE J941 characterizes possible eye locations using a three-dimensional statistical distribution known as the eyellipse. A similar concept is adopted in ISO 4513, which defines the eyellipse as a statistical representation of driver eye locations for vehicle visibility design and evaluation [8]. These frameworks reflect variations in driver anthropometry and seating position and demonstrate that driver visibility cannot, in general, be represented by a single universal eye point.

Empirical studies have further investigated the relationship between driver eye position and vehicle geometry. Dupuis et al. [9] estimated driver eye heights using a camera-based roadside measurement system and reported an average eye height of approximately 1.27 m, corresponding to an average ratio of approximately 0.82 relative to vehicle height. Capaldo et al. [10] analyzed visibility from the driver’s seat for different passenger-car segments and reported representative geometric relationships between the driver’s eye position and the front of the vehicle. More recently, Mueller et al. [11] developed a camera-based perspective-transformation method for quantifying direct visibility around passenger vehicles and examined the effects of seat position and eye height on the resulting visibility maps. Their results further demonstrate that driver-eye configuration can influence the spatial extent of visible and occluded regions.

Vehicle structural characteristics also influence direct visibility independently of eye position. Matsui and Oikawa [12] experimentally examined pedestrian visibility during turning maneuvers and demonstrated that vehicle structures such as A-pillars can temporarily obscure pedestrians from the driver’s direct view. Epstein et al. [4] analyzed forward blind zones across multiple redesign generations of popular passenger cars, SUVs, and pickup trucks and found substantial changes in near-field outward visibility, highlighting the relationship between evolving vehicle geometry and driver blind zones. These findings indicate that direct visibility is jointly determined by driver-eye configuration and vehicle geometry.

In the present study, detailed driver-seat and eye-location information is unavailable in the TUMTraf annotations. Therefore, the driver eye height and longitudinal eye-to-front distance are defined using representative values derived from previous empirical studies [9,10], rather than being treated as vehicle-specific measurements. This assumption enables a controlled geometric analysis but also constitutes an important limitation, particularly because changes in eye position and frontal obstruction geometry can affect the estimated blind-spot extent.

### 2.2. Geometric and Volumetric Blind-Spot Analysis

Vehicle blind spots have been evaluated using various geometric, experimental, and digital-modeling approaches. Early geometric approaches characterize visibility through the relationship between a driver’s eye point, obstructing vehicle structures, and the nearest visible region surrounding the vehicle. More detailed approaches extend these ground-plane relationships into three-dimensional space. Marshall et al. [13] developed a volumetric projection technique for digitally evaluating a driver’s field of view, addressing limitations associated with visibility measures defined only on a two-dimensional ground plane. Summerskill et al. [2] subsequently applied volumetric projection within a digital human modeling environment to identify blind spots around large goods vehicles, demonstrating that vehicle geometry can substantially affect the size and spatial distribution of regions hidden from the driver’s direct view.

Digital vehicle models have also been used to investigate the effects of vehicle design on vulnerable-road-user visibility. Summerskill and Marshall [14] evaluated a truck concept using digital human modeling and quantified how vehicle-design modifications could improve the driver’s ability to directly observe vulnerable road users. Summerskill et al. [15] further quantified direct-vision performance in terms of visible spatial volume and related these measurements to regulatory approaches for heavy vehicles. Frampton and Millington [16] examined vulnerable-road-user protection using direct- and indirect-vision aids and showed that blind regions around heavy vehicles remain an important safety concern even when supplementary sensing systems are employed. These studies establish a clear connection between vehicle geometry, direct visibility, and vulnerable-road-user safety.

Recent studies have also sought more accessible methods for quantitatively measuring vehicle blind zones. Drake et al. [17] developed and validated a camera-based application for estimating vehicle blind zones and nearest visible points, enabling blind-zone measurements without specialized digital vehicle models. Mueller et al. [11] subsequently proposed a perspective-transformation-based approach for generating direct-visibility maps around passenger vehicles. These approaches demonstrate that blind-zone geometry can be quantified using representations substantially simpler than complete computer-aided design (CAD) models, although they still require vehicle-specific image acquisition and a controlled driver or camera viewpoint.

The safety relevance of such visibility metrics has also received increasing empirical support. Hu and Cicchino [18] analyzed police-reported pedestrian crashes together with direct-visibility measurements of passenger vehicles and found that larger driver-side blind zones, longer frontal nearest-visible-point distances, and narrower frontal fields of view were associated with increased risk of left-turn pedestrian crashes. This evidence reinforces the importance of quantitatively characterizing vehicle-induced blind regions rather than treating them solely as qualitative design characteristics.

Geometric occlusion modeling has additionally been investigated in automated-driving sensor evaluation. Genser et al. [19] proposed a geometric occlusion model for virtual testing of advanced driver-assistance and automated-driving functions, demonstrating that simplified three-dimensional object representations can support spatial reasoning about field of view and occlusion. Although the sensing viewpoint and intended application differ from driver direct-vision analysis, such work demonstrates the broader utility of compact geometric object representations for spatial occlusion modeling.

Collectively, these studies show that detailed CAD- and digital-human-modeling approaches can provide high-fidelity representations of direct visibility, whereas camera-based and simplified geometric approaches can provide more scalable estimates with reduced geometric detail. The present study investigates an intermediate representation: GT 3D bounding boxes are used to retain vehicle-specific dimensions and spatial pose while avoiding the requirement for complete CAD geometry and driver accommodation models.

Table 1 summarizes the differences in required inputs, estimated outputs, and validation approaches between representative visibility-assessment methods and the proposed framework.

### 2.3. Vehicle Geometry from 2D and 3D Traffic Perception Data

Computer-vision-based traffic perception provides another source of vehicle information for large-scale road-scene analysis. Conventional image-based object detectors primarily represent vehicles using two-dimensional bounding boxes and class labels. Although these representations are suitable for vehicle detection and classification, they do not directly provide metric vehicle dimensions, depth, or three-dimensional orientation. Monocular 3D object detection methods attempt to recover such information from images, but depth and scale remain intrinsically challenging because three-dimensional geometry must be inferred from two-dimensional observations [5].

In contrast, LiDAR-based and multimodal perception systems explicitly represent surrounding objects in three-dimensional space. Large-scale autonomous-driving benchmarks such as KITTI [20], nuScenes [21], and the Waymo Open Dataset [22] have accelerated research on 3D object detection by providing sensor measurements together with annotated 3D bounding boxes. Surveys of 3D object detection consistently identify object location, dimensions, and orientation as fundamental outputs of the 3D perception task [23,24]. These datasets and methods have consequently established the 3D bounding box as a standard compact representation of object geometry and pose in intelligent-vehicle perception [25].

Most early 3D perception benchmarks were collected primarily using vehicle-mounted sensors. More recent datasets have extended 3D perception to roadside and infrastructure viewpoints [26]. Ye et al. introduced Rope3D, a large-scale roadside perception dataset containing 3D object annotations for infrastructure-mounted camera views [27]. Yu et al. introduced DAIR-V2X for vehicle-infrastructure cooperative perception, providing camera and LiDAR measurements with 3D annotations and benchmarks for infrastructure-side and cooperative 3D object detection [28]. Zimmer et al. developed the TUMTraf Intersection dataset for roadside camera-LiDAR perception at a complex urban intersection [29]. Together, these datasets demonstrate that metric object dimensions, positions, and orientations can be obtained and benchmarked from infrastructure-based sensing environments rather than only from ego-vehicle platforms.

Despite these advances, 3D bounding-box annotations in traffic perception datasets are predominantly used as reference information for training and evaluating perception tasks such as 3D object detection, localization, and tracking. Their use as compact geometric inputs for downstream driver-visibility analysis has received comparatively limited attention. Conversely, detailed direct-vision studies generally rely on CAD geometry, physical vehicle measurements, driver accommodation models, or vehicle-specific imagery that is not normally included in roadside perception datasets.

This distinction leaves a methodological gap between two research domains. Traffic-perception research provides scalable vehicle-level 3D geometry and pose but typically focuses on perception accuracy [30], whereas direct-visibility research provides detailed blind-zone assessments but often requires richer vehicle- and driver-specific information. The present study investigates whether GT 3D bounding boxes can serve as an intermediate geometric representation connecting these domains. Rather than proposing a new 3D object detector, vehicle-specific dimensions, positions, and orientations provided by TUMTraf are used to isolate and evaluate the geometric blind-spot modeling stage. This formulation also establishes a controlled GT-based reference for future work in which estimated 3D detections can replace GT annotations to examine how perception errors propagate to downstream blind-spot estimates.

## 3. Materials and Methods

### 3.1. Dataset and Vehicle-Level Data Preparation

#### 3.1.1. TUMTraf Dataset

This study uses the R2_S1 and R2_S3 sequences from the TUMTraf Release R2 dataset, which contains complex traffic scenarios recorded at a busy urban intersection [29]. TUMTraf was acquired using roadside camera and LiDAR sensors and provides synchronized multimodal data together with 3D object annotations. Unlike conventional 2D image annotations, the TUMTraf annotations provide metric 3D bounding-box information, including the center coordinates, length, width, height, object class, and quaternion-based orientation of each annotated object. These attributes enable vehicle-specific geometric information and spatial pose to be incorporated directly into the subsequent blind-spot analysis.

The 3D annotations are provided in JSON files following the OpenLABEL format. In addition, TUMTraf provides calibration information between the LiDAR and camera sensors, allowing 3D points defined in the LiDAR coordinate system to be transformed into the corresponding camera coordinate system and projected onto the image plane. This calibration information is used in this study for the spatial validation described in Section 3.4. The R2_S1 and R2_S3 sequences contain 564 and 2066 frames, respectively, and no missing calibration information was identified in the frames used in this study.

#### 3.1.2. Vehicle-Level Annotation Aggregation

Because the same vehicle may appear in multiple consecutive frames, the frame-level annotations were aggregated to construct a vehicle-level representation. Individual objects were initially identified using the combination of sequence_id and object_id, and annotations sharing the same identifier within a sequence were treated as observations of the same object.

Because the North and South LiDAR annotations are generated independently in TUMTraf, the same physical vehicle may be assigned different object identifiers across the two sensor sources. Therefore, a direct comparison of object identifiers is insufficient to identify cross-LiDAR duplicate observations. To account for this issue, object annotations from the two LiDAR sources were transformed into a common coordinate system using the coordinate-transformation information provided with the dataset. Potential cross-LiDAR duplicates were identified using a multistage verification procedure. After transforming the annotations from both LiDAR sensors into the common s110_base coordinate system, North and South LiDAR frames were matched by the nearest timestamp within a tolerance of 0.06 s. Object pairs whose 3D centers were within 1.0 m were retained as initial candidates. Candidate pairs were then evaluated using temporal overlap, spatial proximity, trajectory direction, vehicle dimensions, class consistency, and the number of temporally aligned observations.

The temporal-overlap ratio was calculated from the observation intervals of the North and South LiDAR tracks. Let $t_{N}^{\mathrm{start}}$ and $t_{N}^{\mathrm{end}}$ denote the earliest and latest timestamps of the North LiDAR track, respectively, and let $t_{S}^{\mathrm{start}}$ and $t_{S}^{\mathrm{end}}$ denote those of the South LiDAR track. The temporal-overlap ratio was defined as

(1) $$R_{\mathrm{overlap}}=\frac{\max\left(0,\min\left(t_{N}^{\mathrm{end}},t_{S}^{\mathrm{end}}\right)-\max\left(t_{N}^{\mathrm{start}},t_{S}^{\mathrm{start}}\right)\right)}{\min\left(t_{N}^{\mathrm{end}}-t_{N}^{\mathrm{start}},t_{S}^{\mathrm{end}}-t_{S}^{\mathrm{start}}\right)}.$$

The denominator represents the shorter of the two track observation durations, rather than the longer duration or the union of the observation intervals. The ratio was computed using continuous timestamp-based durations in seconds, not the number of matched frames. For a zero-duration track, the ratio was defined as zero.

A pair was automatically classified as a confirmed duplicate when the temporal-overlap ratio was at least 0.5, at least 80% of the aligned observations had a center distance within 1.0 m, the mean trajectory-direction cosine similarity was at least 0.8, the median length, width, and height differences did not exceed 0.30, 0.15, and 0.20 m, respectively, the object classes matched, and at least 10 aligned observations were available. Trajectory direction was evaluated only when at least five inter-frame displacements exceeded 0.05 m to avoid unreliable direction estimates for nearly stationary vehicles.

Pairs that could not be confidently classified by these rules were retained as uncertain and visually inspected by overlaying the North and South GT 3D bounding boxes on the corresponding camera images. Among 47 initial candidate pairs, 14 were automatically confirmed, 10 were classified as uncertain, and 23 were classified as non-duplicates. Visual inspection identified three additional duplicates among the uncertain pairs, resulting in 17 confirmed duplicate pairs. Because tracking discontinuities could assign multiple object identifiers to the same physical vehicle, the confirmed pairs were grouped into connected components using a Union-Find procedure. Within each component, the observation with the largest number of tracked frames was retained; when frame counts were equal, the observation with the larger number of LiDAR points was retained. This procedure removed 12 duplicate entries, reducing the preliminary set from 230 to 218 CAR instances.

The analysis was restricted to the CAR class to examine vehicle-specific geometric variation under a common set of representative visibility parameters. This controlled setting allows the effects of GT vehicle width and spatial pose to be investigated without introducing additional differences arising from class-specific driver-eye positions and frontal obstruction geometries.

Class consistency was also examined at the vehicle level. The initial class aggregation yielded 230 preliminary CAR instances from 231 identified objects. In R2_S3, five object identifiers contained both CAR and VAN labels across different frames. These ambiguous cases were examined using both the frame-level class-label distribution and visual inspection of the corresponding vehicles by projecting their GT 3D bounding boxes onto the camera images. This reexamination revealed a tracking discontinuity in which two object identifiers, separated by 0.099 s and 0.0385 m in 3D position, corresponded to the same physical vehicle. Among the remaining ambiguous cases, two objects that were predominantly labeled as CAR were visually identified as VANs and were therefore reclassified. One additional object was visually identified as a pickup truck; because the TUMTraf class taxonomy used in this study does not provide a corresponding pickup-truck category, this object was excluded from the CAR analysis.

The subsequent class reexamination excluded three additional vehicles from the 218 CAR instances remaining after cross-LiDAR duplicate removal, resulting in 215 representative CAR instances, comprising 41 vehicles from R2_S1 and 174 from R2_S3.

To reduce frame-to-frame variation in the annotated vehicle geometry, representative length, width, and height values were calculated for each vehicle as the medians of the corresponding frame-level annotations. Representative center coordinates were similarly obtained by taking the median independently along each coordinate axis. For vehicle orientation, the circular nature of yaw angles was explicitly considered: each frame-level yaw angle was first converted to a unit direction vector, the component-wise medians of these vectors were calculated, and the resulting representative direction was converted back to a yaw angle using the atan2 function. The resulting representative yaw values were additionally checked against the corresponding camera images to verify that they were consistent with the observed vehicle orientation.

The resulting median dimensions and center coordinates, together with the validated representative yaw, were used for vehicle-level geometric analysis. For the camera-based qualitative spatial validation, however, the center coordinates and GT yaw from the specific frame being projected were used to preserve spatial correspondence with the camera image. This frame-specific pose representation accounts for actual changes in vehicle heading during turning maneuvers, which may not be fully captured by a vehicle-level representative yaw.

#### 3.1.3. Geometric Validity Criteria

Before applying the frontal blind-spot model, the 215 representative CAR instances obtained through the vehicle-level data preparation procedure were screened for geometric validity. As an initial data integrity check, vehicles were considered invalid if the required GT 3D bounding-box information contained missing or non-finite values or if the representative length, width, or height was nonpositive. No vehicle was excluded according to these criteria.

Three additional criteria were imposed to ensure geometric consistency with the bounding-box-based approximation adopted in this study. First, the frontal obstruction height $h_{f}$ was required to be lower than the GT vehicle height *H* ($h_{f}<H$), ensuring that the assumed obstruction point lies within the vertical extent of the bounding box.

Second, the driver eye height $h_{e}$ was required to satisfy $h_{e}\leq H$. This condition provides a basic consistency check by requiring the assumed driver eye point to remain within the vertical extent of the bounding-box representation. It does not, however, establish the actual driver-eye location within the vehicle cabin.

Third, the longitudinal eye-to-front distance *d* was required to satisfy d<L, where *L* denotes the representative GT vehicle length. This criterion ensures that the assumed longitudinal eye position remains within the front-to-rear extent of the bounding-box representation.

Among the 215 representative CAR instances, one vehicle had a representative GT height of approximately 0.995 m, which was lower than the assumed driver eye height of 1.27 m. Inspection of its camera images further indicated that the GT bounding box did not adequately encompass the visible vehicle body. Consequently, this vehicle was excluded from the geometric analysis.

The remaining 214 CAR instances satisfied the adopted validity criteria under all three frontal-obstruction-height scenarios ($h_{f}=0.965$, 1.030, and 1.160 m). The same 214 vehicles were therefore retained for the baseline analysis, sensitivity analysis, and mean-width comparison to ensure consistency across the experimental conditions.

### 3.2. Frontal Blind-Spot Geometric Model

The proposed model combines vehicle-specific geometric information from the TUMTraf GT 3D bounding boxes with representative driver-visibility parameters to quantify the frontal blind spot. For each vehicle, the GT annotation provides the bounding-box center $C={\left(x_{c},y_{c},z_{c}\right)}^{T}$, length *L*, width *W*, height *H*, and yaw angle ψ. These quantities are used together with three visibility parameters: the driver eye height $h_{e}$, the longitudinal eye-to-front distance *d*, and the frontal obstruction height $h_{f}$. The model first determines the frontal blind-spot distance *D* from the side-view geometry and then uses the vehicle-specific width *W* to quantify the corresponding ground area *A* and spatial volume *V*. The resulting geometry is subsequently placed in the road coordinate system using the vehicle position and orientation, as described in Section 3.3.

#### 3.2.1. Model Assumptions and Visibility Parameters

Because the TUMTraf annotations do not provide vehicle-specific driver seat locations, eye positions, or detailed front-end surface geometry, the proposed model adopts representative visibility parameters rather than treating these quantities as vehicle-specific measurements. Table 2 summarizes the three parameters used in the baseline geometric model.

The driver eye height $h_{e}$ was set to 1.27 m based on the roadside measurements reported by Dupuis et al. [9]. The longitudinal distance *d* from the ground projection of the driver eye point to the frontal reference point was set to 2.00 m based on the geometric relationship reported by Capaldo et al. [10]. The frontal obstruction height $h_{f}$ was set to 1.03 m as the representative baseline front-end height adopted in this study. The baseline value of $h_{f}=1.030$ m was used as a reference configuration for the controlled geometric analysis, rather than as an empirically established obstruction height applicable to all CAR instances. Because vehicle-specific frontal obstruction measurements are unavailable in TUMTraf, the absolute estimates obtained under this setting are conditional on the assumed parameter value. The effects of alternative obstruction-height assumptions are therefore examined through the sensitivity analyses described in Section 3.5.1. These three parameters were held constant across all CAR instances in the baseline analysis.

For geometric tractability, the driver eye point was assumed to lie on the vehicle longitudinal centerline, corresponding to a lateral eye offset of $s_{e}=0$. This assumption defines a symmetric reference configuration rather than representing the actual driver-seat position of individual vehicles. Because TUMTraf does not provide vehicle-specific seat locations or detailed frontal obstruction geometry, the present study adopts this simplified configuration to isolate the effects of GT vehicle width and spatial pose.

In actual vehicles, the driver’s eye is laterally displaced from the centerline, and the resulting visibility boundaries may be asymmetric. Accurately modeling such asymmetry would require vehicle-specific lateral eye positions and frontal obstruction boundaries, which are not available in the dataset. Therefore, the estimated blind-spot regions should be interpreted as symmetric geometric approximations rather than complete representations of actual driver visibility.

#### 3.2.2. Blind-Spot Distance

**Figure 1:**
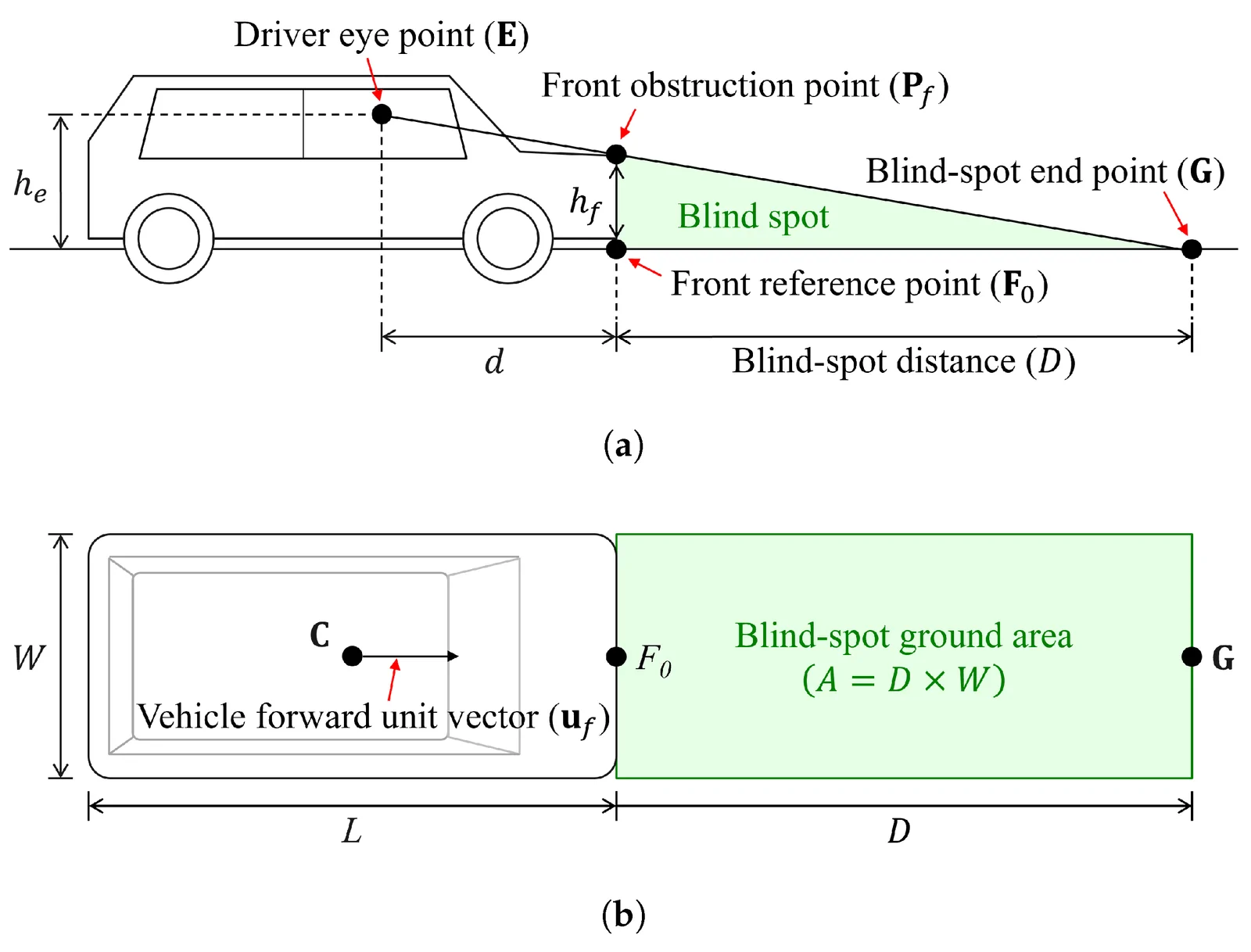
Geometric representation of the proposed frontal blind-spot model: (**a**) side-view geometry defining the driver eye point E, frontal obstruction point $P_{f}$, frontal reference point $F_{0}$, blind-spot endpoint G, and blind-spot distance *D*; and (**b**) plan-view representation of the rectangular ground-plane footprint with length *D* and vehicle-specific width *W*. The three-dimensional blind-spot region is approximated by extending the triangular region in (**a**) across the width shown in (**b**), forming a triangular prism.

The frontal blind-spot distance is derived from the side-view geometry illustrated in Figure 1a. Let $F_{0}$ denote the frontal reference point on the ground plane. The driver eye point E is located a longitudinal distance *d* behind $F_{0}$ and at a height $h_{e}$ above the ground, whereas the frontal obstruction point $P_{f}$ is located vertically above $F_{0}$ at a height $h_{f}$. The line of sight passing from E through $P_{f}$ intersects the ground plane at G. The longitudinal distance from $F_{0}$ to G defines the frontal blind-spot distance *D*.

From the two similar triangles formed by the line of sight, $d/\left(h_{e}-h_{f}\right)=D/h_{f}$. Accordingly, the frontal blind-spot distance is given by

(2) $$D=d\frac{h_{f}}{h_{e}-h_{f}},$$

where the model requires $h_{e}>h_{f}$.

Because $h_{e}$, *d*, and $h_{f}$ are held constant across all CAR instances within a given scenario, *D* is also constant across vehicles in that scenario. Vehicle-to-vehicle variation in the estimated blind-spot area and volume therefore arises from the vehicle-specific GT width *W*, as described in Section 3.2.3. In the frontal-obstruction-height-only sensitivity analysis, $h_{f}$ is varied while $h_{e}$ and *d* are held constant. A separate joint sensitivity analysis examines the combined effects of $h_{e}$, *d*, and $h_{f}$, as described in Section 3.5.1.

#### 3.2.3. Blind-Spot Area and Volume

In addition to the longitudinal blind-spot distance, the proposed model quantifies the ground-plane extent and three-dimensional spatial extent of the frontal blind spot. As illustrated in Figure 1b, the ground-plane footprint is approximated as a rectangular region extending a distance *D* forward from the frontal reference point and spanning the vehicle-specific GT width *W*. This rectangular footprint represents only the vehicle-width corridor extending longitudinally in front of the vehicle. The lateral extent is assumed to remain constant at the GT vehicle width *W* along the entire distance *D*. Accordingly, the model does not explicitly trace lateral sightlines from the driver eye point or estimate the full lateral boundaries of the physically occluded region. The blind-spot ground area *A* is therefore defined as

(3) A=DW.

To represent the corresponding three-dimensional occluded region, the side-view triangular region bounded by the ground plane, the frontal obstruction height $h_{f}$, and the line of sight from the driver’s eye point through the frontal obstruction point is extended across the vehicle width *W*. The resulting region is approximated as a triangular prism. Accordingly, the blind-spot volume *V* is defined as

(4) $$V=\frac{1}{2}Dh_{f}W.$$

Under the adopted model, *D* is determined exclusively by the representative visibility parameters $h_{e}$, *d*, and $h_{f}$, whereas *W* is obtained from the GT 3D bounding box of each vehicle. Because the visibility parameters are held constant across vehicles within a given scenario, vehicle-to-vehicle variation in *A* and *V* within that scenario is determined solely by the vehicle-specific width *W*. This distinction is important when interpreting the proposed framework: the GT width contributes to the magnitude of the estimated ground area and volume, whereas the remaining GT 3D bounding-box information is used primarily for spatial placement of the resulting geometry, as described in Section 3.3.

### 3.3. GT 3D Bounding-Box-Based Spatial Placement

The geometric quantities defined in Section 3.2 describe the size of the frontal blind spot but do not, by themselves, specify its location or orientation in the road coordinate system. To spatially place the estimated blind-spot geometry, the GT 3D bounding-box center $C={\left(x_{c},y_{c},z_{c}\right)}^{T}$, length *L*, height *H*, and yaw angle ψ are used to determine the vehicle front and forward direction.

The vehicle forward unit vector in the ground plane is defined as

(5) $$u_{f}=\left[\begin{array}{c} \cos\psi \\ \sin\psi \\ 0 \end{array}\right].$$

Because the GT bounding-box center is defined at the geometric center of the box, the ground-level frontal reference point $F_{0}$ is obtained by moving from the bounding-box center downward by H/2 and forward by L/2:

(6) $$F_{0}=\left[\begin{array}{c} x_{c}+\frac{L}{2}\cos\psi \\ y_{c}+\frac{L}{2}\sin\psi \\ z_{c}-\frac{H}{2} \end{array}\right].$$

The vertical coordinate $z_{c}-H/2$ is used as a local ground reference under the assumption that the bottom of the GT 3D bounding box approximately coincides with the road surface beneath the vehicle. This approximation is consistent with the yaw-only bounding-box representation adopted in the present framework.

However, it does not explicitly account for local road-surface variation along the estimated blind-spot region, vehicle pitch and roll, or errors in the annotated bounding-box height and vertical position. Consequently, the estimated ground-plane footprint should be interpreted as a simplified locally planar representation rather than an exact reconstruction of the road surface.

Let $e_{z}={\left[0,0,1\right]}^{T}$ denote the vertical unit vector. Using $F_{0}$ as the common spatial reference, the driver eye point E, frontal obstruction point $P_{f}$, and ground-level blind-spot endpoint G are positioned as

(7) $$\begin{array}{cc} E & =F_{0}-du_{f}+h_{e}e_{z}, \end{array}$$

(8) $$\begin{array}{cc} P_{f} & =F_{0}+h_{f}e_{z}, \end{array}$$

(9) $$\begin{array}{cc} G & =F_{0}+Du_{f}. \end{array}$$

Under the lateral-centerline assumption introduced in Section 3.2.1, E and $P_{f}$ lie in the same longitudinal vertical plane as $F_{0}$. The vehicle-specific GT width *W* is then applied symmetrically about this centerline to construct the lateral boundaries of the rectangular ground-plane footprint and the corresponding triangular-prism volume illustrated in Figure 1b.

Through this transformation, the blind-spot distance, ground area, and volume defined in Section 3.2 are not treated only as scalar quantities; the corresponding geometric region is positioned according to the vehicle-specific GT location and orientation in the road coordinate system. The GT width determines the lateral extent of the region, whereas the bounding-box center, length, height, and yaw determine its spatial reference and orientation.

### 3.4. Calibration-Based Camera Projection

To visually examine the spatial consistency of the GT 3D bounding boxes and estimated frontal blind-spot geometries with the corresponding camera images, the official TUMTraf sensor calibration information was used.

Unlike the cross-LiDAR duplicate verification procedure in Section 3.1.2, which transforms annotations into the common s110_base coordinate system, camera projection was performed directly in the coordinate system of the North or South LiDAR that provided the corresponding frame-level annotation. The GT bounding box and blind-spot geometry were constructed using the frame-specific coordinates in that LiDAR reference frame, without an intermediate transformation through s110_base.

For each LiDAR–camera pair, the corresponding 3×4 projection matrix *P* was selected according to the annotation’s sensor identifier. The matrix incorporates the LiDAR-to-camera extrinsic transformation and camera intrinsic parameters, expressed as P=K[R∣t], where *R* and *t* denote the extrinsic rotation and translation, respectively, and *K* denotes the camera intrinsic matrix. The official projection matrix was applied directly to the homogeneous LiDAR coordinates.

Let $P_{L}={\left[x_{L},y_{L},z_{L}\right]}^{T}$ denote a 3D point expressed in the native coordinate system of the LiDAR sensor identified by the corresponding frame-level annotation. The projection matrix *P* is selected for that specific LiDAR–camera pair, ensuring that the input 3D coordinates and calibration matrix refer to the same LiDAR reference frame. Using the homogeneous representation of $P_{L}$, the corresponding homogeneous image coordinates are obtained as

(10) $$\left[\begin{array}{c} \tilde{u}\\ \tilde{v}\\ \tilde{w} \end{array}\right]=P\left[\begin{array}{c} x_{L}\\ y_{L}\\ z_{L}\\ 1 \end{array}\right].$$

The corresponding pixel coordinates are then obtained through homogeneous normalization:

(11) $$u=\frac{\tilde{u}}{\tilde{w}},\;v=\frac{\tilde{v}}{\tilde{w}}.$$

Four projection matrices provided by TUMTraf were used, corresponding to all combinations of the North and South LiDAR sensors and the South1 and South2 cameras.

The same projection procedure was applied to the vertices of the GT 3D bounding box and to the geometric elements defining the estimated frontal blind spot. For camera-based validation, the vehicle dimensions (length, width, and height) were obtained from the vehicle-level median GT values calculated across the corresponding track, whereas the GT center coordinates and yaw angle were taken from the specific frame selected for projection. Thus, the vehicle-level median dimensions were retained while the frame-specific pose was used to preserve spatial correspondence with the camera image, particularly for vehicles undergoing turning maneuvers.

The same combination of vehicle-level median dimensions and frame-specific GT pose was consistently applied to both the GT 3D bounding-box projection and the estimated frontal blind-spot geometry.

When a vehicle was observable from more than one available camera, the GT 3D bounding box was projected into each candidate image. Among valid projections, the camera view providing the largest projected GT bounding-box area within the image was selected for visualization. The blind-spot geometry was then projected onto the selected image using the same calibration parameters. This procedure provides a common image-space representation of the vehicle GT geometry and the estimated blind-spot region and forms the basis of the qualitative spatial validation described in Section 3.5.

### 3.5. Validation Protocol

Three complementary validation procedures were designed to examine different aspects of the proposed geometric framework. First, sensitivity analyses were conducted to examine the effects of the representative visibility parameters on the estimated blind-spot geometry. These included a frontal-obstruction-height-only analysis and a joint sensitivity analysis of driver eye height, longitudinal eye-to-front distance, and frontal obstruction height.

Second, a mean-width baseline comparison was performed to quantify the information loss caused by replacing vehicle-specific GT widths with a single representative width. Third, calibration-based camera projection was used to qualitatively assess the spatial consistency of the estimated blind-spot placement with the corresponding vehicle position and orientation. The three procedures are described below.

#### 3.5.1. Sensitivity Analysis of Visibility Parameters

Because the frontal obstruction height $h_{f}$ is not directly available from the TUMTraf annotations, its influence on the estimated blind-spot geometry was examined through sensitivity analysis. The driver eye height $h_{e}$ and longitudinal eye-to-front distance *d* were fixed at 1.27 m and 2.00 m, respectively, while $h_{f}$ was varied across three illustrative scenarios: 0.965 m, 1.030 m, and 1.160 m.

These values represent lower, baseline, and higher frontal-obstruction-height assumptions, respectively, rather than vehicle-specific measurements of actual visibility obstruction points. The scenarios were selected to examine the sensitivity of the simplified geometric model to plausible variations in the assumed front-end obstruction height. They do not represent a reclassification of the TUMTraf CAR instances into sedan, CAR, or SUV body types.

For each $h_{f}$ scenario, the blind-spot distance *D*, ground area *A*, and volume *V* were recalculated using the geometric model defined in Section 3.2. Following the geometric validity screening described in Section 3.1.3, the same 214 CAR instances were used in all three scenarios. This common analysis population ensures that differences in the resulting blind-spot estimates arise solely from changes in $h_{f}$, rather than from differences in the included vehicles.

In addition to the three representative scenarios, $h_{f}$ was varied continuously from 0.965 m to 1.160 m in increments of 0.005 m while holding $h_{e}$ and *d* constant. This analysis was used to characterize the nonlinear response of *D* as $h_{f}$ approaches $h_{e}$, as implied by (2). The resulting sensitivity curve and scenario-specific blind-spot statistics are presented in Section 4.2.

To further examine the combined effects of the representative visibility parameters, a joint sensitivity analysis was conducted by varying $h_{e}$, *d*, and $h_{f}$ simultaneously. Three values were considered for each parameter: $h_{e}\in \left\{1.17,1.27,1.37\right\}$ m, d∈{1.80,2.00,2.20} m, and $h_{f}\in \left\{0.965,1.030,1.160\right\}$ m, resulting in 27 parameter combinations.

For each combination, the geometric validity criteria ($h_{f}<H$, $h_{e}\leq H$, and d<L) were applied independently to the 215 representative CAR instances. The blind-spot distance *D*, mean ground area *A*, mean volume *V*, and number of valid vehicles were then calculated for each configuration.

Configurations satisfying $0<h_{e}-h_{f}<0.02$ m were separately identified as near-singular cases because the denominator of the distance equation approaches zero. These cases were retained to illustrate the mathematical sensitivity of the model but were not interpreted as physically validated blind-spot estimates.

#### 3.5.2. Mean-Width Baseline Comparison

A controlled baseline comparison was conducted to quantify the information loss introduced when vehicle-specific width information is replaced with a single representative width. For each geometrically valid CAR instance under the baseline parameter setting ($h_{f}=1.03$ m), the vehicle-specific GT width $W_{i}$ was used in the proposed formulation, whereas the baseline formulation replaced $W_{i}$ with the mean width $\bar{W}$ calculated across all valid CAR instances. All other geometric parameters and assumptions were held unchanged. Thus, the comparison isolates the effect of replacing vehicle-specific width information with a category-level representative value.

For vehicle *i*, the absolute width error $AE_{i}$ and percentage width error $PE_{i}$ were defined as

(12) $$AE_{i}=\left|\bar{W}-W_{i}\right|,\;PE_{i}=\frac{\left|\bar{W}-W_{i}\right|}{W_{i}}\times 100\%.$$

The corresponding mean absolute error (MAE) and mean absolute percentage error (MAPE) across the *N* valid vehicles were calculated as

(13) $$\mathrm{MAE}=\frac{1}{N}\sum_{i=1}^{N}\left|\bar{W}-W_{i}\right|,$$

(14) $$\mathrm{MAPE}=\frac{100}{N}\sum_{i=1}^{N}\frac{\left|\bar{W}-W_{i}\right|}{W_{i}}.$$

Because the blind-spot ground area and volume are linear functions of vehicle width under a fixed geometric scenario, the absolute differences caused by the mean-width approximation were calculated as

(15) $$\Delta A_{i}=D\left|\bar{W}-W_{i}\right|,$$

and

(16) $$\Delta V_{i}=\frac{1}{2}Dh_{f}\left|\bar{W}-W_{i}\right|.$$

The relative errors in *A* and *V* are mathematically identical to the relative width error because the common multiplicative terms cancel. Accordingly, separate MAPE values for width, ground area, and volume were not treated as independent performance measures. The comparison was therefore interpreted as an assessment of geometric information loss caused by the mean-width approximation rather than as an accuracy evaluation against ground-truth blind-spot measurements.

#### 3.5.3. Qualitative Spatial Validation

A camera-projection-based validation was conducted to assess whether the estimated blind-spot geometry was spatially consistent with the corresponding vehicle position and orientation in the road scene. This validation focused on the spatial placement of the geometric model rather than on the quantitative accuracy of the estimated blind-spot distance, area, or volume, for which image-based ground truth was not available.

An initial projection attempt using a single representative frame per vehicle produced valid camera projections for 90 of the 214 geometrically valid CAR instances. Because reliance on a single frame can exclude vehicles that are outside the camera field of view or poorly visible in that particular frame, the projection search was subsequently expanded across the available frames in which each previously uncovered vehicle appeared and across the available calibrated camera views. For each candidate frame–camera combination, the GT 3D bounding box and the corresponding blind-spot geometry were projected using the calibration procedure described in Section 3.4. A vehicle was included in the expanded projection pool when a frame–camera combination allowed the relevant projected geometry to be visually assessed within the image. The expanded search increased the number of projectable vehicles from 90 to 172 out of the 214 valid CAR instances, and these 172 vehicles formed the candidate pool for the subsequent qualitative validation.

The initial validation sample comprised 30 vehicles, including five purposively selected illustrative cases and 25 additional cases selected through width-stratified random sampling. The five illustrative cases represented near-range, far-range, narrow-width, wide-width, and partially occluded conditions.

The 25 additional cases were originally selected using width-stratified random sampling with a fixed random seed of 20260826. However, a subsequent audit identified differences between the preliminary sampling candidate pool and the final vehicle-level analysis population, including inconsistencies in the width values used for stratum assignment.

Reconstructing the sampling procedure using the final representative GT widths and the final projectable pool resulted in a different set of selected vehicles. Therefore, the original validation sample was retained as an exploratory sample rather than being treated as a reproducible stratified random sample of the final projectable population.

The qualitative validation results are reported only for the retained evaluated cases and are not intended to estimate spatial-validity prevalence across the full CAR population.

Subsequent cross-LiDAR duplicate and class-consistency reexamination excluded three selected objects: two cross-LiDAR duplicate observations and one object with inconsistent CAR/VAN labels. No replacement cases were introduced, leaving 27 vehicles in the final qualitative validation sample.

To assess the spatial consistency of the projected blind-spot geometry, each selected case was evaluated using five predefined criteria: (1) frontal reference point placement relative to the vehicle front, (2) consistency of the projected forward direction with the observed vehicle orientation, (3) placement of the estimated blind-spot region in front of the vehicle, (4) visibility of the calibration-based projection, and (5) interpretability of the vehicle and blind-spot geometry within the road scene.

Each criterion was assessed using a five-level qualitative scale: appropriate, mostly appropriate, partially appropriate, inappropriate, or undeterminable. An overall verdict was also assigned using the same scale based on the combined assessment of the five criteria.

The overall verdict was assigned independently by each assessor through a holistic visual judgment of the projected geometry, taking all five assessment criteria into account. No numerical aggregation, weighting, averaging, or majority-voting rule was applied to combine the individual criterion ratings.

In making the overall judgment, assessors considered the severity of any observed spatial discrepancies and their influence on the interpretability and plausibility of the projected blind-spot placement. The overall verdict was recorded separately from the five criterion-level ratings.

For reporting purposes, the overall verdicts were grouped into four categories: Valid (appropriate), Partially Valid (mostly appropriate or partially appropriate), Invalid (inappropriate), and Undeterminable (insufficient visual information for a reliable assessment). Undeterminable cases were retained in the evaluation denominator but were not counted as Valid or Partially Valid. Partially Valid was used when some spatial discrepancies were observed but the overall placement remained interpretable and was not judged clearly inappropriate. Undeterminable was assigned when insufficient visual information, such as substantial occlusion or very small projected vehicle size, prevented a reliable judgment.

The assessment focused on the spatial consistency and visual interpretability of the projected geometry rather than on the physical accuracy of the estimated blind-spot distance, area, or volume.

To assess the reproducibility of the qualitative judgments, the 27 sampled cases were evaluated by three assessors: two original study authors and one additional assessor who had not participated in the original evaluation. The reassessment used the final camera projections generated with frame-specific GT yaw and the same predefined assessment criteria.

The two original assessors repeated the evaluation with vehicle-selection metadata concealed, while the additional assessor independently evaluated the same cases without access to the original verdicts. For each assessor, the counts of Valid, Partially Valid, Invalid, and Undeterminable outcomes were recorded separately.

Inter-assessor agreement was evaluated using pairwise percentage agreement and Cohen’s kappa for each of the five assessment criteria and the overall verdict. Fleiss’ kappa was also calculated to summarize agreement among all three assessors for the overall verdict after mapping the original five-level scale to the four reporting categories.

Among the 27 vehicles retained for qualitative spatial validation, five representative cases were selected for detailed visualization. These cases illustrate five characteristic observation conditions: near range, narrow vehicle width, wide vehicle width, partial occlusion, and far range.

All five illustrative cases were included in the final 27-vehicle validation sample and were therefore also evaluated under the same multi-assessor assessment protocol. The representative cases are presented separately for visualization purposes, whereas the quantitative summary of qualitative assessment outcomes is based on all 27 evaluated vehicles. The corresponding results and projection examples are presented in Section 4.4.

## 4. Results

### 4.1. Overall Vehicle and Blind-Spot Characteristics

Under the baseline parameter setting ($h_{e}=1.27$ m, d=2.00 m, and $h_{f}=1.03$ m), 214 of the 215 representative CAR instances satisfied the geometric validity criteria and were included in the primary analysis. The remaining vehicle, with a representative GT height of approximately 0.995 m, failed to satisfy both $h_{f}<H$ and $h_{e}\leq H$. Camera-image inspection further indicated an incomplete GT bounding-box representation, supporting its exclusion from the analysis.

Table 3 summarizes the vehicle-width distribution and the corresponding frontal blind-spot characteristics of the 214 valid CAR instances. The mean GT vehicle width was 1.902 m, with a standard deviation of 0.170 m, and the widths ranged from 1.376 to 2.626 m. Under the baseline visibility parameters, the frontal blind-spot distance *D* was 8.583 m for all valid vehicles.

The resulting blind-spot ground area had a mean of 16.328 m^2^ and a standard deviation of 1.459 m^2^, with values ranging from 11.809 to 22.538 m^2^. The corresponding blind-spot volume had a mean of 8.409 m^3^ and a standard deviation of 0.751 m^3^, ranging from 6.082 to 11.607 m^3^. Because the visibility parameters and, consequently, *D* were fixed under the baseline setting, the observed vehicle-to-vehicle variation in ground area and volume reflects the variation in vehicle-specific GT width.

### 4.2. Sensitivity Analysis Results

**Figure 2:**
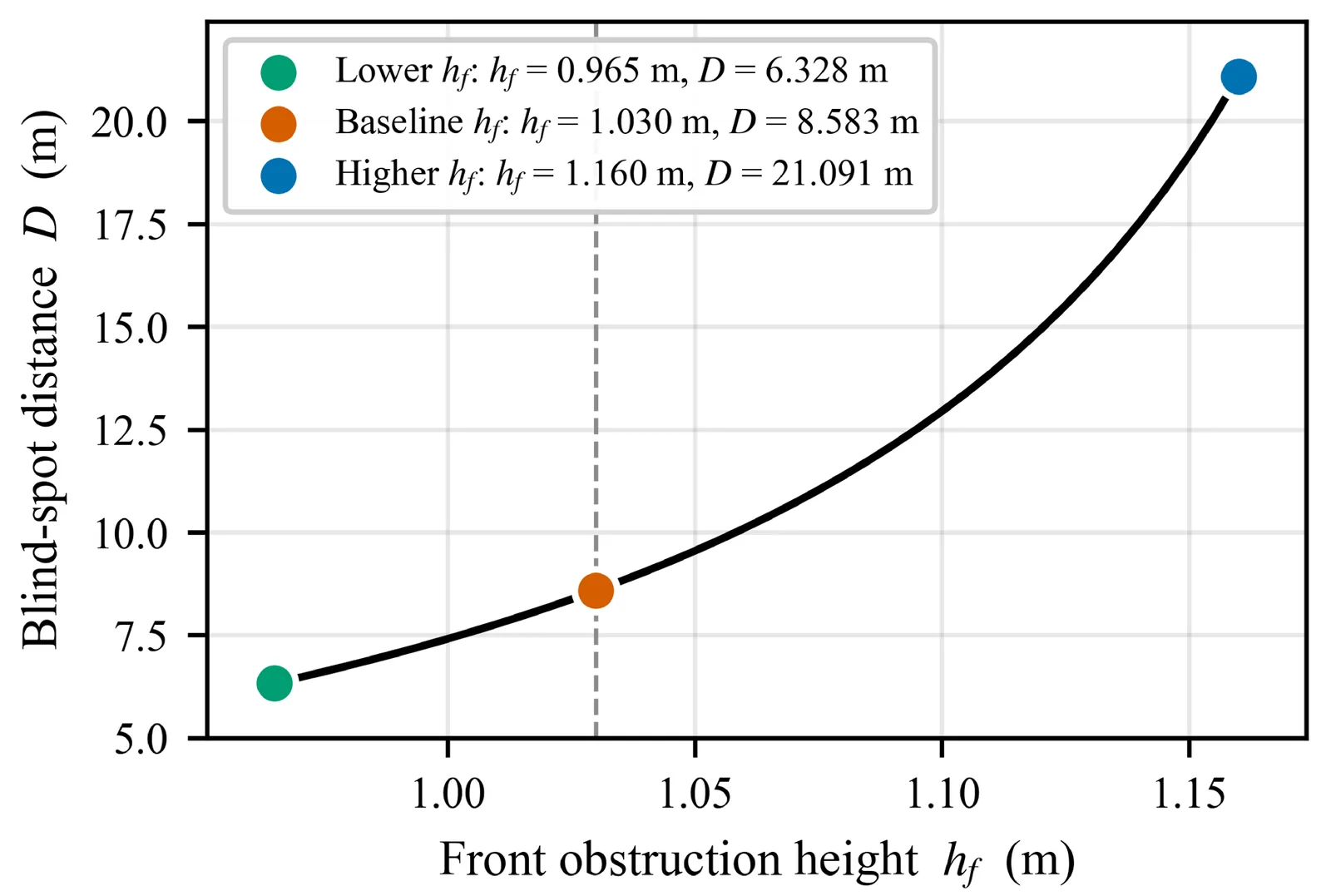
Sensitivity of the frontal blind-spot distance *D* to the frontal obstruction height $h_{f}$, with $h_{e}=1.27$ m and d=2.00 m held constant. The markers indicate the lower, baseline, and higher $h_{f}$ scenarios evaluated in Table 4.

Figure 2 shows the nonlinear relationship between the frontal obstruction height $h_{f}$ and the frontal blind-spot distance *D*, while Table 4 summarizes the results for the three representative $h_{f}$ scenarios. In the lower-$h_{f}$ scenario ($h_{f}=0.965$ m), *D* was 6.328 m, corresponding to a 26.3% decrease relative to the baseline value of 8.583 m. In contrast, increasing $h_{f}$ from the baseline value of 1.030 m to 1.160 m increased *D* to 21.091 m, corresponding to a 145.7% increase.

This pronounced increase results from the nonlinear dependence of *D* on $h_{f}$ in (2). As $h_{f}$ increases from 1.030 to 1.160 m, the denominator $h_{e}-h_{f}$ decreases from 0.240 to 0.110 m. Consequently, *D* increases rapidly as $h_{f}$ approaches the fixed driver eye height $h_{e}=1.27$ m. The local sensitivity of *D* to $h_{f}$ is given by

(17) $$\frac{\partial D}{\partial h_{f}}=\frac{dh_{e}}{{\left(h_{e}-h_{f}\right)}^{2}}.$$

At the baseline setting, this derivative is approximately 44.1, indicating that a 0.01-m increase in $h_{f}$ around the baseline value corresponds to an increase of approximately 0.44 m in *D*.

The geometric validity screening described in Section 3.1.3 excluded one vehicle whose representative GT height was approximately 0.995 m, which was lower than the assumed driver eye height of 1.27 m. Consequently, the same 214 CAR instances were retained for all three frontal-obstruction-height scenarios. This common analysis population allows the effects of varying $h_{f}$ to be compared without changes in vehicle composition.

The changes in *D* propagated directly to the absolute ground-area and volume estimates. Using the common set of 214 CAR instances, the mean blind-spot ground area increased from 12.038 m^2^ in the lower-$h_{f}$ scenario to 16.328 m^2^ under the baseline setting and 40.122 m^2^ in the higher-$h_{f}$ scenario. The corresponding mean volumes were 5.808, 8.409, and 23.271 m^3^, respectively. Because *D* is constant across vehicles within each scenario, vehicle-to-vehicle variation in these quantities remains determined by the GT width, while changes in $h_{f}$ scale their absolute magnitudes.

Although $h_{f}$ strongly affected the absolute values of *D*, *A*, and *V*, it did not affect the relative error introduced by the mean-width approximation. For the ground area,

(18) $$\frac{\left|A_{i}^{\mathrm{GT}}-A_{i}^{\mathrm{mean}}\right|}{A_{i}^{\mathrm{GT}}}=\frac{\left|DW_{i}-D\bar{W}\right|}{DW_{i}}=\frac{\left|W_{i}-\bar{W}\right|}{W_{i}},$$

so the common factor *D* cancels. The same cancellation occurs for the volume because both formulations contain the common factor $\frac{1}{2}Dh_{f}$. Because the same 214 CAR instances and the same mean GT width were used across all three scenarios, the relative errors introduced by the mean-width approximation were identical across the scenarios, consistent with the cancellation of the common geometric factors. Thus, the assumed frontal obstruction height substantially affects the absolute scale of the estimated blind spot but not the relative information loss associated with replacing vehicle-specific GT widths with the mean-width baseline.

**Figure 3:**
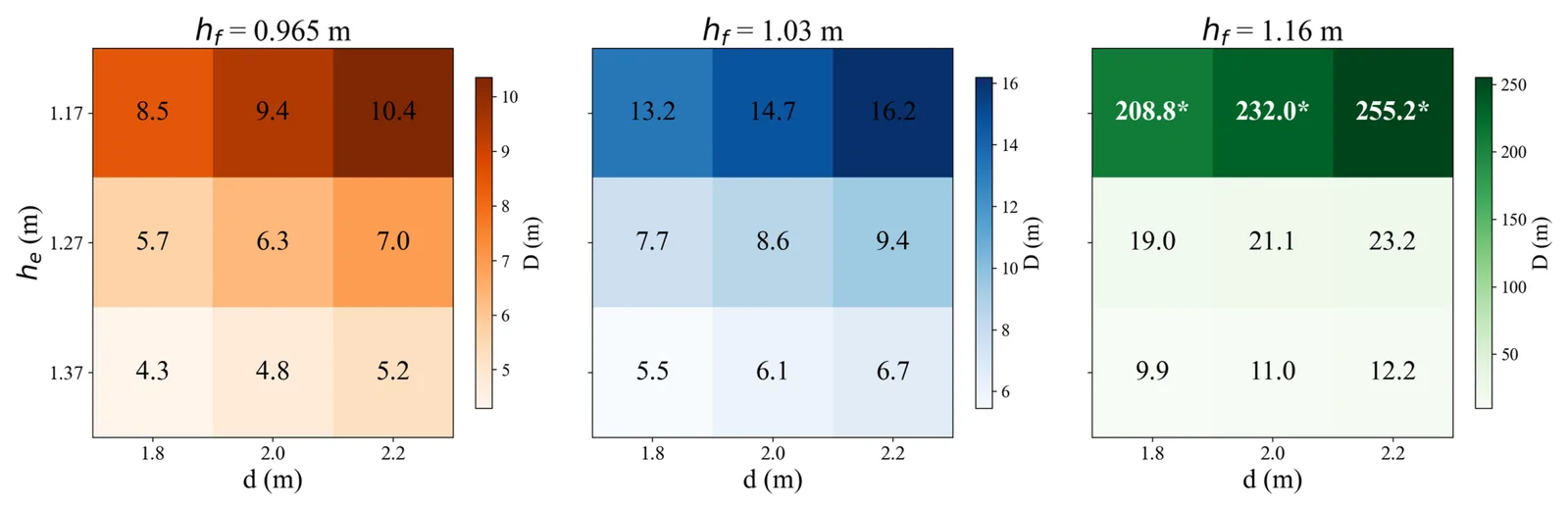
Joint sensitivity of the estimated frontal blind-spot distance *D* to the driver eye height $h_{e}$, longitudinal eye-to-front distance *d*, and frontal obstruction height $h_{f}$. The three panels correspond to $h_{f}=0.965$, 1.030, and 1.160 m, respectively. Each panel uses an independent color scale; therefore, numerical annotations should be used for cross-panel comparisons. The white asterisks identify near-singular configurations ($0<h_{e}-h_{f}<0.02$ m), which are retained to illustrate model sensitivity rather than physical visibility measurements.

The joint sensitivity analysis further examined the combined effects of driver eye height $h_{e}$, eye-to-front distance *d*, and frontal obstruction height $h_{f}$ across 27 parameter combinations. Figure 3 summarizes the resulting blind-spot distances.

The geometric validity screening retained 214 vehicles for configurations with $h_{e}=1.17$ or 1.27 m and 213 vehicles for configurations with $h_{e}=1.37$ m. Among the 27 combinations, 24 produced blind-spot distances ranging from 4.289 to 23.200 m. The remaining three configurations, characterized by $h_{e}=1.17$ m and $h_{f}=1.160$ m, yielded substantially larger distances of 208.8, 232.0, and 255.2 m for d=1.80, 2.00, and 2.20 m, respectively.

These three configurations satisfy $h_{e}-h_{f}=0.01$ m and were therefore identified as near-singular cases. Their unusually large distances reflect the mathematical behavior of the geometric model as $h_{f}$ approaches $h_{e}$, rather than physically validated frontal blind-spot measurements.

Across the remaining configurations, increasing $h_{f}$ or *d* increased the estimated distance, whereas increasing $h_{e}$ reduced it. These results demonstrate that the absolute blind-spot estimates depend on the combined visibility assumptions rather than on frontal obstruction height alone.

### 4.3. Mean-Width Baseline Comparison Results

Table 5 summarizes the quantitative effects of replacing the vehicle-specific GT widths with the mean-width baseline. Across the 214 valid CAR instances, the mean GT width was 1.902 m. The mean absolute error between the vehicle-specific GT widths and the mean-width baseline was 0.108 m, corresponding to a MAPE of 5.8%. The maximum relative width error reached 38.3%, indicating that the approximation error was substantially larger for some vehicles whose widths differed markedly from the sample mean.

Because the blind-spot ground area and volume depend linearly on vehicle width under the fixed baseline geometry, the width differences propagated directly to the corresponding blind-spot estimates. The mean absolute difference in ground area was 0.923 m^2^, with a maximum difference of 6.210 m^2^. For blind-spot volume, the mean and maximum absolute differences were 0.476 and 3.198 m^3^, respectively.

**Figure 4:**
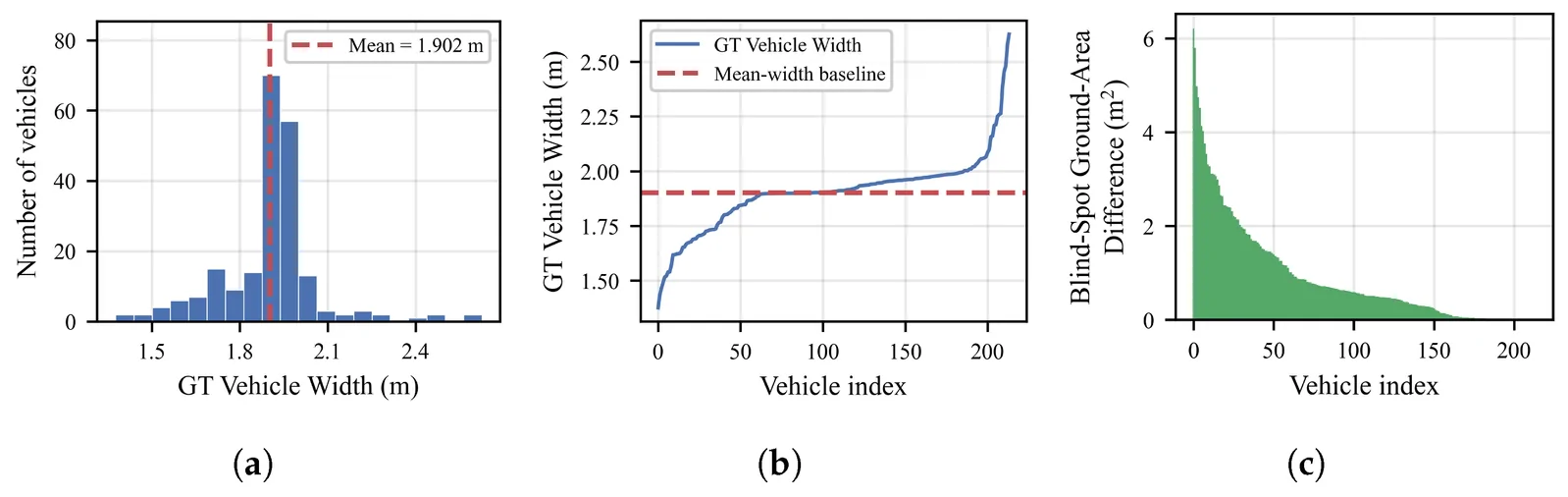
Effect of the mean-width baseline approximation on vehicle-specific GT width and blind-spot ground area (n=214): (**a**) distribution of GT vehicle widths, with the dashed line indicating the mean width of 1.902 m; (**b**) vehicle-specific GT widths sorted in ascending order and compared with the constant mean-width baseline; and (**c**) absolute difference in blind-spot ground area sorted in descending order. Panels (**b**,**c**) are independently sorted; therefore, the same vehicle index does not necessarily refer to the same vehicle in both panels.

Figure 4 further illustrates the effect of the mean-width approximation. Figure 4a shows the distribution of vehicle-specific GT widths and their mean, whereas Figure 4b compares the individual GT widths, sorted in ascending order, with the constant mean-width baseline. Figure 4c shows the resulting absolute differences in blind-spot ground area. Vehicles with widths close to the sample mean produced relatively small differences, whereas larger deviations from the mean width resulted in correspondingly larger differences in the estimated ground area.

To assess the robustness of the mean-width comparison to potentially questionable bounding-box annotations, the projected GT boxes of vehicles with extreme widths were further inspected. Two instances with GT widths of 2.482 and 2.251 m exhibited noticeable spatial mismatches between their projected bounding boxes and the visible vehicle contours. These instances were therefore excluded in an additional robustness analysis, while the original 214-vehicle population was retained for the primary results.

After excluding the two instances, the mean GT width changed from 1.902 to 1.898 m, and the width MAE decreased from 0.108 to 0.105 m. The MAPE changed from 5.8% to 5.7%, while the mean absolute differences in blind-spot ground area and volume changed from 0.923 to 0.902 m^2^ and from 0.476 to 0.464 m^3^, respectively. The maximum relative width error remained similar (38.3% versus 38.0%).

Although the maximum absolute area and volume differences increased slightly after recomputing the mean-width baseline, the overall results remained largely unchanged. This robustness check suggests that the main findings of the controlled mean-width comparison are not driven solely by these two questionable bounding-box projections.

### 4.4. Qualitative Spatial Validation Results

The multi-frame, multi-camera projection search increased the projectable vehicle pool from 90 to 172 of the 214 geometrically valid CAR instances. The qualitative validation sample was originally constructed from a preliminary candidate pool comprising five purposively selected illustrative cases and 25 additional cases selected through width-stratified sampling.

Subsequent data reexamination excluded three selected objects that did not belong to the final valid CAR population, leaving 27 vehicles for qualitative assessment. A retrospective audit identified inconsistencies between the preliminary sampling pool and the final projectable population, including differences in the width values used for stratum assignment.

Accordingly, the retained 27 cases are treated as an exploratory validation sample rather than a reproducible stratified random sample of the final projectable population. The following qualitative results describe only these evaluated cases and are not intended to provide population-level estimates.

As summarized in Table 6, the three assessors independently evaluated the 27 sampled vehicles using the predefined qualitative criteria. Assessor 1 classified 12 vehicles as Valid, 12 as Partially Valid, and three as Undeterminable. Assessor 2 classified 17 as Valid, seven as Partially Valid, one as Invalid, and two as Undeterminable. The additional independent assessor classified 14 as Valid, ten as Partially Valid, one as Invalid, and two as Undeterminable.

All three assessors classified 24 of the 27 vehicles (88.9%) as either Valid or Partially Valid. However, the distribution between these two categories differed across assessors, indicating variability in the qualitative judgments. The reported 88.9% therefore represents a consistent aggregate proportion across the three assessors rather than complete agreement on individual vehicle classifications.

For the four-category overall verdict, pairwise agreement was 74.1% between the independent assessor and Assessor 1 (Cohen’s κ=0.566), 74.1% between the independent assessor and Assessor 2 (κ=0.546), and 66.7% between Assessors 1 and 2 (κ=0.441).

Agreement among all three assessors was moderate, with Fleiss’ κ=0.512. All three assessors assigned the same overall category to 16 of the 27 vehicles (59.3%), whereas at least one assessor disagreed for the remaining 11 vehicles. These disagreements primarily concerned the distinction between adjacent assessment categories rather than opposing judgments of clearly valid and invalid spatial placement.

At the criterion level, pairwise agreement varied across the five assessment dimensions. Blind-spot position showed consistently high percentage agreement (88.9% for all assessor pairs), whereas frontal reference point placement showed greater variability, with agreement ranging from 44.4% to 81.5%.

Agreement for yaw direction ranged from 63.0% to 70.4%, camera projection from 59.3% to 63.0%, and interpretability from 66.7% to 81.5%. These results indicate that some aspects of spatial placement were evaluated more consistently than others, while subjective image-based judgments remained a source of variability.

**Figure 5:**
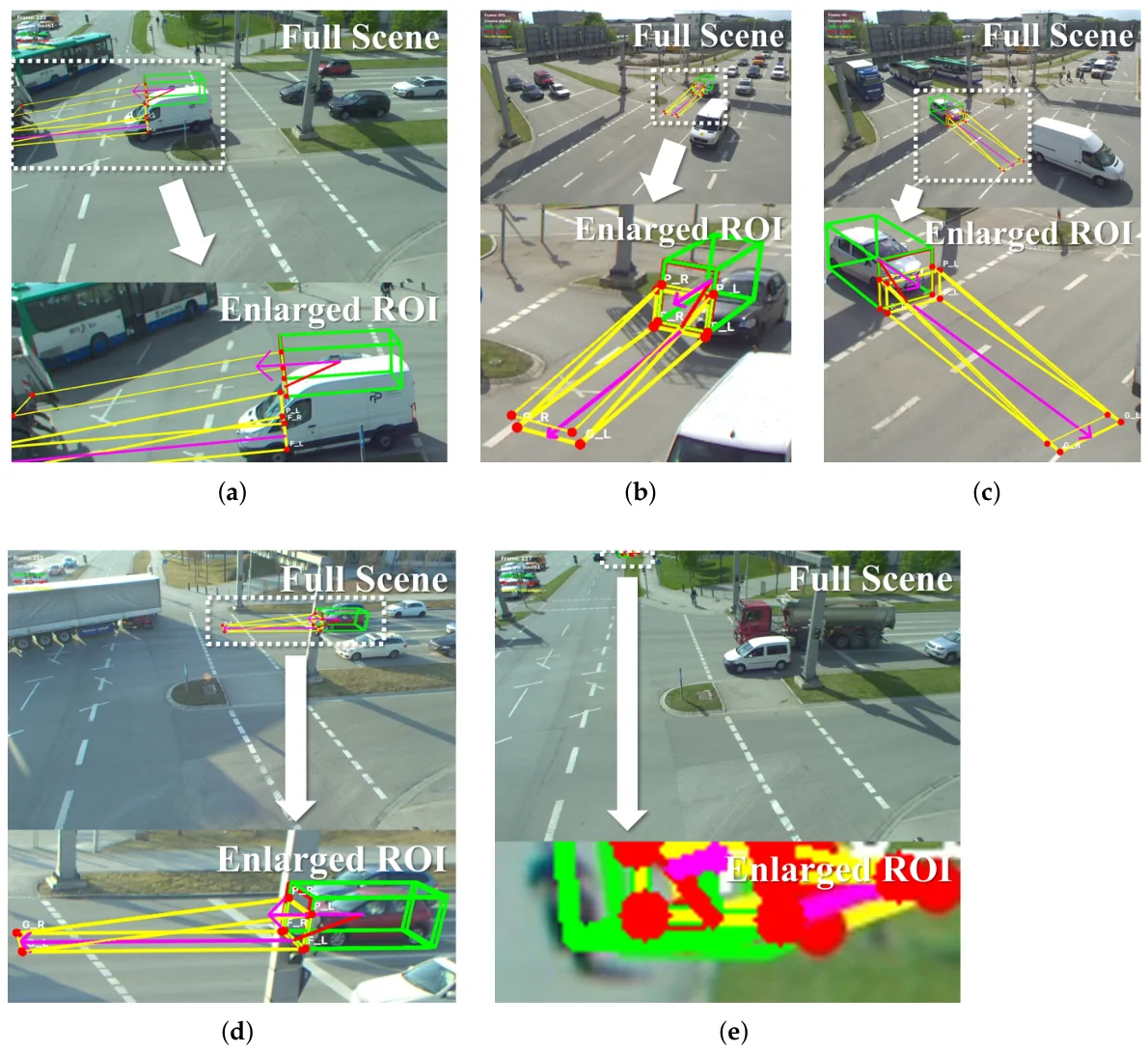
Representative camera-projection examples under five observation conditions: (**a**) near range, (**b**) narrow vehicle width, (**c**) wide vehicle width, (**d**) partial occlusion, and (**e**) far range. The overlays show the GT 3D bounding box (green), frontal reference point (red), vehicle forward direction (magenta), and estimated frontal blind-spot geometry (yellow). Enlarged regions of interest (ROIs) are provided to facilitate inspection of the frontal reference points, forward directions, and blind-spot boundaries.

Figure 5 presents five representative projection examples selected to illustrate different observation conditions. In the near-range example (Figure 5a), the frontal reference point and yaw-based forward direction were consistent with the observed vehicle, and the estimated blind-spot region was positioned in front of the vehicle. Part of the projected blind-spot geometry extended beyond the camera field of view, illustrating a limitation of image-based visual assessment rather than a failure of the geometric calculation.

The narrow- and wide-width examples (Figure 5b,c) correspond to GT widths of 1.376 and 2.626 m, respectively, representing the minimum and maximum widths among the 214 valid CAR instances. In both cases, the frontal reference point, forward direction, and blind-spot placement were visually consistent with the corresponding vehicle. For the wide-width example, the projected GT 3D bounding box showed some spatial margin relative to the visible vehicle contour; therefore, the projection was interpreted in terms of identifiable position and orientation rather than exact contour agreement.

In the partially occluded example (Figure 5d), foreground objects limited visual assessment of the frontal reference region, although the vehicle forward direction and approximate blind-spot placement remained interpretable. In contrast, the far-range example (Figure 5e) was clipped near the image boundary, preventing reliable visual assessment of the frontal reference point and blind-spot placement. This case was retained explicitly as a limitation example rather than being interpreted as a failure of the blind-spot calculation itself.

A reassessment of the five representative cases with the vehicle selection metadata concealed showed complete agreement for the criteria directly related to spatial placement, namely the yaw-based forward direction and blind-spot location, when evaluated using the three-level positive/intermediate/negative categorization. In contrast, agreement for more subjective criteria, including the overall assessment and projection quality, was 40%. No case changed between opposite assessment categories; the observed differences reflected the degree of assessment rather than a reversal between appropriate and inappropriate placement. These findings indicate greater consistency for the directly geometry-based criteria than for the more subjective image-based assessments.

To examine the influence of vehicle orientation on camera-based spatial validation, the projections of all 27 sampled vehicles were regenerated using the frame-specific GT yaw instead of the vehicle-level representative yaw. In both configurations, the center coordinates corresponded to the selected projection frame.

The angular difference between the two yaw representations had a mean of 1.12 degrees, a median of 0.003 degrees, and a maximum of 11.58 degrees. Eight vehicles exhibited angular differences greater than 1 degree, including two with differences greater than 5 degrees.

Visual comparison of the regenerated projections showed noticeable changes in the blind-spot boundaries for the turning vehicle with the largest yaw difference. However, the updated geometry remained spatially consistent with the observed vehicle orientation and road scene. For the remaining vehicles, the changes were comparatively small.

The reassessment of the regenerated projections did not change any of the 27 overall qualitative verdicts. Consequently, the proportion classified as Valid or Partially Valid remained 24/27 (88.9%). These results indicate that frame-specific yaw better preserves instantaneous vehicle heading during turning maneuvers without changing the aggregate spatial-validity result in the evaluated sample.

## 5. Discussion

### 5.1. Implications of Vehicle-Specific Geometric Information

The results demonstrate two distinct roles of vehicle-specific GT 3D bounding-box information in the proposed framework: quantitative representation of vehicle-width variation and spatial placement of the resulting blind-spot geometry. Under a fixed set of visibility parameters, the frontal blind-spot distance *D* is identical across vehicles, whereas the ground area *A* and volume *V* vary in direct proportion to the vehicle-specific GT width *W*. Consequently, the mean-width baseline comparison provides a controlled measure of the geometric information lost when individual vehicle widths are replaced by a single category-level representative value.

The mean-width baseline produced a MAPE of 5.8% relative to the vehicle-specific GT widths, while the maximum relative width error reached 38.3%. These differences propagated directly to the estimated blind-spot ground area and volume because both quantities depend linearly on *W*. Importantly, these results should not be interpreted as an improvement in blind-spot estimation accuracy, because no vehicle-specific ground-truth measurements of the actual blind spots were available for comparison. Rather, the results quantify the extent to which a mean-width approximation suppresses the geometric variation present in the GT 3D bounding boxes. The relatively large maximum error further indicates that a category-level representative width can substantially misrepresent vehicles located toward the extremes of the observed width distribution, even when the average error across the sample is moderate.

The remaining GT 3D bounding-box attributes serve a different purpose. The bounding-box center, length, height, and yaw do not introduce vehicle-to-vehicle variation in *D*, *A*, or *V* under the present fixed-parameter formulation. Instead, they provide the spatial reference required to position and orient the estimated blind-spot geometry in the road coordinate system. In particular, the center and length determine the frontal reference location, the height establishes the ground-level reference of the bounding-box approximation and is also used in the geometric validity check, and yaw determines the forward direction of the vehicle. The calibration-based camera projections provide complementary evidence that this spatial information can be used to associate the estimated blind-spot geometry with the corresponding vehicle position and orientation in the road scene.

This distinction is central to interpreting the contribution of the proposed framework. The use of GT 3D bounding boxes does not imply that all components of the bounding box independently determine the magnitude of the estimated blind spot. Rather, vehicle-specific width preserves variation in the estimated ground area and volume, while the remaining geometric and pose information enables the resulting region to be spatially anchored to an individual vehicle. In this sense, the GT 3D bounding box provides a compact intermediate representation between category-level vehicle dimensions, which discard individual geometric variation and pose, and detailed CAD-based visibility models, which require substantially richer vehicle and driver information.

The practical contribution of this framework is therefore not an improvement in the physical accuracy of direct-visibility estimation, but the integration of simplified blind-spot quantification with vehicle-specific spatial placement. Unlike scalar estimates based solely on representative vehicle dimensions, the proposed approach uses GT bounding-box pose information to associate each estimated blind-spot region with an individual vehicle in a common road coordinate system. This provides a compact intermediate representation that can support future roadside perception applications without requiring complete vehicle CAD models.

### 5.2. Sensitivity to Visibility Assumptions

The sensitivity analysis demonstrates that the absolute magnitude of the estimated frontal blind spot depends strongly on the assumed visibility parameters, particularly the frontal obstruction height $h_{f}$. Increasing $h_{f}$ from the baseline value of 1.030 m to 1.160 m increased the frontal blind-spot distance *D* from 8.583 to 21.091 m, despite the relatively modest change in $h_{f}$. This behavior is inherent to the geometric formulation because *D* increases nonlinearly as $h_{f}$ approaches the fixed driver eye height $h_{e}$. Accordingly, the absolute values of *D*, *A*, and *V* should be interpreted in relation to the assumed driver-visibility parameters rather than as vehicle-specific measurements of actual blind spots.

The joint sensitivity analysis further demonstrates that the estimated frontal blind-spot distance is strongly influenced by the combined assumptions regarding driver eye height, eye-to-front distance, and frontal obstruction height. Among the 27 parameter combinations, 24 yielded distances between 4.289 and 23.200 m, whereas three near-singular configurations produced substantially larger values of 208.8–255.2 m. These extreme results arise when $h_{e}$ approaches $h_{f}$, causing the denominator of the geometric formulation to approach zero.

Although the near-singular configurations satisfy the adopted bounding-box-based validity criteria, their resulting distances should not be interpreted as physically realistic measurements of frontal blind spots. Rather, they reveal a fundamental limitation of the simplified geometric model: small differences between the assumed eye height and obstruction height can lead to disproportionately large changes in the estimated blind-spot distance. Consequently, reliable vehicle-specific estimates would require better-constrained visibility parameters and independent physical validation.

The geometric validity assessment also highlighted the importance of ensuring consistency between the assumed driver-eye position and the GT vehicle dimensions. One CAR instance had a representative GT height of approximately 0.995 m, which was lower than the assumed driver eye height of 1.27 m. Camera-image inspection further indicated that its GT bounding box did not adequately encompass the visible vehicle body. Excluding this instance and using the same 214 vehicles across the three frontal-obstruction-height scenarios with $h_{e}=1.27$ m held constant eliminated changes in sample composition and enabled a controlled comparison of the effects of varying $h_{f}$. In the separate joint sensitivity analysis, the validity criteria were applied independently to each parameter combination, resulting in 213 or 214 valid vehicles depending on the assumed driver eye height. Nevertheless, the adopted validity criteria ensure only basic consistency within the bounding-box approximation and do not establish the actual driver-eye position or physical visibility geometry.

This sensitivity is particularly relevant because $h_{f}$ is not provided by the TUMTraf annotations and is therefore represented by an assumed value in the present framework. The same consideration applies, although not identically, to the representative driver eye height $h_{e}$ and longitudinal distance *d*, which are also not vehicle-specific measurements. Consequently, the GT 3D bounding boxes provide vehicle-specific geometric and pose information, but they do not eliminate uncertainty associated with the driver viewpoint and the detailed frontal structure of the vehicle. This distinction defines an important boundary of the present GT-based formulation.

Importantly, the sensitivity of the absolute blind-spot magnitude to $h_{f}$ does not invalidate the controlled comparison between vehicle-specific GT widths and the mean-width baseline. As shown in Section 4.2, the common geometric terms cancel when the relative difference between the two width formulations is calculated. The relative information loss caused by replacing $W_{i}$ with $\bar{W}$ is therefore independent of the assumed value of $h_{f}$ under the present model. The sensitivity analysis and the mean-width comparison consequently address two different aspects of the framework: the former characterizes uncertainty in the absolute scale of the estimated blind spot, whereas the latter quantifies the loss of vehicle-specific width information under a controlled set of geometric assumptions.

These findings suggest that improving the estimation of vehicle-specific visibility parameters is an important direction for extending the proposed framework. In particular, replacing a fixed frontal obstruction height with vehicle-specific measurements or estimates could allow the blind-spot distance itself, rather than only the width-dependent ground area and volume, to vary according to individual vehicle geometry. Such an extension would move the framework toward a more complete vehicle-specific visibility model while retaining the spatial information available from roadside 3D perception data.

### 5.3. Spatial Validation and Practical Interpretation

The calibration-based camera projection provides complementary evidence for the spatial component of the proposed framework [31,32,33]. By extending the projection search across multiple frames and calibrated camera views, the number of projectable vehicles increased from 90 to 172 of the 214 valid CAR instances. This expanded coverage reduced the dependence of the validation on a single representative frame and enabled the subsequent qualitative assessment to include a broader range of vehicle widths and observation conditions.

The reported spatial-validity proportion of 88.9% (24/27) describes only the evaluated qualitative validation sample. It should not be interpreted as an estimate of geometric accuracy or spatial-validity prevalence across all 214 valid CAR instances. Moreover, the retrospective sampling audit identified discrepancies between the preliminary candidate pool and the final projectable population. Consequently, the retained sample cannot be regarded as a reproducible stratified random sample of the final projectable population.

The validation sample was selected partly through purposive case selection and partly through width-stratified random sampling. Furthermore, 42 vehicles were excluded from the projectable pool because no evaluated frame–camera combination satisfied the projection-visibility requirements. These sampling and coverage constraints limit the generalizability of the qualitative assessment.

In the final sampled validation set, 24 of 27 vehicles (88.9%) were classified as Valid or Partially Valid according to the predefined spatial-assessment criteria. This result supports the feasibility of using GT 3D bounding-box position and orientation information to place the estimated blind-spot geometry in correspondence with individual vehicles in the road scene. However, the 88.9% result should not be interpreted as the accuracy of the blind-spot estimation itself. The camera images do not provide ground-truth measurements of the actual blind-spot distance, ground area, or volume; therefore, the projection analysis evaluates the plausibility and spatial consistency of the geometric placement rather than the quantitative correctness of its magnitude.

The representative projection cases further illustrate both the capability and the observational limitations of the validation procedure. Near-range and width-extreme cases allowed the frontal reference point, vehicle forward direction, and blind-spot placement to be interpreted directly from the projected geometry. In partially occluded scenes, some geometric elements became difficult to assess visually, while far-range objects could be clipped by the image boundary and therefore become unsuitable for reliable image-based assessment. These cases indicate that the interpretability of the projection depends not only on the geometric formulation but also on camera field of view, object distance, occlusion, and the visibility of the projected geometry in the selected image.

The comparison between vehicle-level representative yaw and frame-specific GT yaw highlights the importance of preserving instantaneous vehicle orientation during camera-based spatial validation. Although the two yaw representations were similar for most sampled vehicles, noticeable differences were observed in some turning scenarios. Using frame-specific yaw avoids treating genuine heading changes as frame-level annotation noise and provides a more appropriate pose representation for projecting vehicle-specific blind-spot geometry.

In the evaluated sample, regenerating the projections with frame-specific yaw did not change the overall qualitative verdicts, and the aggregate spatial-validity proportion remained 88.9%. Nevertheless, this agreement does not establish the quantitative accuracy of the estimated blind-spot geometry. The comparison evaluates the consistency of spatial placement under alternative orientation representations rather than the correspondence between the estimated blind-spot boundaries and independently measured direct visibility.

The independent reassessment of the 27 sampled vehicles provides additional evidence regarding the reproducibility of the qualitative spatial validation. All three assessors classified 24 of the 27 vehicles (88.9%) as either Valid or Partially Valid. However, agreement on individual four-category verdicts was incomplete, with Fleiss’ κ=0.512 and unanimous agreement for 16 of the 27 cases.

These findings indicate that the aggregate spatial-validity proportion was consistent across assessors, although the distinction between Valid, Partially Valid, Invalid, and Undeterminable remained partly subjective. In particular, differences in the visibility of the frontal reference point, image resolution, occlusion, and projection interpretability may contribute to variation in individual assessments.

The earlier reassessment of the five illustrative cases showed lower agreement for subjective criteria, including overall assessment and projection quality. However, this limited reassessment and the subsequent 27-case multi-assessor evaluation differ in sample size and assessment categorization and should not be interpreted as directly comparable agreement estimates.

Taken together, the results support the feasibility of qualitative spatial validation using camera-projected GT geometry while highlighting the need for more objective assessment criteria and independent physical visibility measurements.

Despite these encouraging spatial-consistency results, camera-based projection does not constitute a substitute for vehicle-specific blind-spot ground truth. A stronger validation would require independent measurements of actual driver visibility or occluded regions for the corresponding vehicles, which are not available in the TUMTraf dataset. The present camera-based assessment should therefore be viewed as a spatial feasibility check that complements, rather than validates the absolute accuracy of, the geometric blind-spot estimates.

If independent ground-truth measurements of physical direct visibility were available, the proposed framework could be evaluated by comparing its estimated frontal blind-spot distance with the measured nearest visible ground point under corresponding driver-eye and vehicle configurations. Such a comparison would enable quantitative assessment of the geometric model, rather than only the spatial consistency of its projected placement.

Discrepancies could arise from the use of representative driver eye height, eye-to-front distance, and frontal obstruction height, as well as the centered-eye assumption and the simplified bounding-box-based front-end geometry. In particular, the sensitivity analysis indicates that uncertainty in the frontal obstruction height can substantially affect the estimated blind-spot distance. Therefore, independent visibility measurements would be necessary to determine the quantitative accuracy and potential systematic biases of the proposed estimates.

As a preliminary assessment of external validation feasibility, we also examined the supplementary field-measurement data reported by Drake et al. [17]. These data provide frontal nearest-visible-point measurements under different seating configurations for a 2006 Chevrolet Suburban, together with selected eye-position-related vehicle dimensions.

However, the supplementary data from that study do not provide an independently measured frontal obstruction height corresponding to $h_{f}$ in the proposed model. Furthermore, the reported distance units and some eye-height values require clarification before a consistent quantitative comparison can be established.

Consequently, these measurements were not used to calculate prediction errors for the proposed geometric model. This limitation reinforces the need for a reference dataset containing matched driver-eye positions, frontal obstruction geometry, and directly measured visibility distances.

### 5.4. Limitations and Future Work

Several limitations should be considered when interpreting the results of this study. First, the TUMTraf annotations do not provide vehicle-specific driver eye positions, seat locations, or detailed front-end geometry. Consequently, the visibility parameters $h_{e}$, *d*, and $h_{f}$ were represented using fixed values rather than vehicle-specific measurements. As demonstrated by the sensitivity analysis, particularly for $h_{f}$, these assumptions can substantially affect the absolute magnitude of the estimated blind-spot distance, ground area, and volume. Future work should therefore incorporate vehicle-specific visibility parameters obtained from direct measurement, vehicle-model information, or suitable estimation methods.

Second, the frontal obstruction point $P_{f}$ is defined on the front plane of the GT 3D bounding box rather than on the actual vehicle surface. The bounding-box representation does not capture detailed front-end geometry such as hood curvature, windshield geometry, or other structural features that may determine the true line-of-sight boundary. In addition, the driver eye point is assumed to lie on the longitudinal centerline of the vehicle ($s_{e}=0$). Actual driver seating positions have a lateral offset from the vehicle centerline, which can produce asymmetric visibility and blind-spot regions between the two sides of the vehicle. Extending the model to incorporate detailed front-end geometry and vehicle- or driving-side-specific lateral eye offsets would enable a more realistic asymmetric representation of direct visibility. Furthermore, the constant-width corridor approximation does not account for the lateral expansion or contraction of actual visibility boundaries. Consequently, the estimated ground area and volume represent simplified geometric quantities within the vehicle-width corridor rather than the complete physically occluded region surrounding the vehicle.

Third, because the same representative values of $h_{e}$, *d*, and $h_{f}$ are applied to all vehicles within each scenario, the frontal blind-spot distance *D* does not vary across vehicles under the present formulation. Vehicle-to-vehicle variation in the estimated ground area and volume is therefore determined by the vehicle-specific GT width *W*. Although the GT bounding-box center, length, height, and yaw enable vehicle-specific spatial placement, the present framework should not be interpreted as a complete vehicle-specific visibility model. Obtaining vehicle-specific frontal obstruction and driver-viewpoint parameters would allow future extensions to introduce individual variation in *D* as well as in the lateral extent of the blind spot.

Fourth, the present analysis was restricted to vehicles annotated as CAR in the TUMTraf dataset to investigate vehicle-specific geometric variation under a common set of representative visibility parameters. Although the dataset also contains VAN, TRUCK, and other vehicle categories, their driver-eye positions and frontal obstruction geometries may differ substantially from those of passenger cars. Furthermore, the CAR category does not distinguish passenger body types such as sedans, hatchbacks, and SUVs. Accordingly, the lower, baseline, and higher frontal-obstruction-height scenarios represent sensitivity conditions rather than empirically assigned body-type categories. Extending the framework to additional vehicle classes would require class-specific visibility parameters, together with consistent annotation verification and vehicle-level preprocessing.

Fifth, the camera-based validation assesses the spatial consistency of the projected geometry rather than the quantitative accuracy of the estimated blind spot. Although the multi-frame and multi-camera projection search increased the number of projectable vehicles, the qualitative evaluation remained limited to 27 sampled cases.

To improve the reproducibility of the assessment, two original assessors and one additional independent assessor evaluated the final frame-specific-yaw projections. All three assessors reported the same aggregate spatial-validity proportion of 88.9%, but agreement on individual four-category verdicts was incomplete (Fleiss’ κ=0.512).

These findings indicate that subjective judgment remains a limitation even when an independent assessor is included. Furthermore, camera-based spatial consistency does not establish the physical accuracy of the estimated blind-spot distance, ground area, or volume. Future validation should therefore incorporate more objective assessment criteria and independent measurements of vehicle-specific direct visibility.

In addition, the cross-LiDAR duplicate verification included single-reviewer visual inspection for ambiguous candidate pairs, and seven uncertain pairs could not be conclusively resolved. Therefore, the possibility of residual cross-sensor duplicate observations cannot be completely excluded.

Finally, the present study intentionally uses GT 3D bounding boxes to isolate the geometric modeling stage from 3D perception errors. Consequently, the reported results do not characterize the performance of a deployable real-time blind-spot estimation system. An important next step is to replace the GT annotations with 3D bounding boxes estimated from roadside camera–LiDAR perception and to quantify how errors in object dimensions, localization, and orientation propagate to the resulting blind-spot geometry. Such an extension would connect the present GT-based geometric framework to practical roadside intelligent-transportation applications.

Another promising direction is to extend the proposed static geometric framework toward dynamic digital twins of roadside traffic environments. By integrating real-time 3D object detection, multi-object tracking, and synchronized roadside sensor observations, vehicle-specific blind-spot geometries could be continuously updated according to changes in vehicle position and orientation.

Such an extension would require addressing uncertainty in estimated 3D bounding boxes, tracking discontinuities, sensor synchronization, and real-time processing latency. Recent studies on adaptive digital-twin model splitting and reliable crowdsourcing [34], federated digital-twin construction through distributed sensing [35], and edge–cloud collaborative updates [36] provide relevant architectural directions for managing distributed sensing and computational resources.

Although the present study does not implement a dynamic digital twin, its GT 3D bounding-box-based geometric quantification and spatial placement provide a potential foundation for future temporally updated blind-spot representations in roadside perception systems.

Security is another important consideration for future deployment in real-time roadside perception environments. Although the present study uses offline GT annotations and does not implement sensor-data transmission or networked processing, practical roadside systems may be exposed to data interception, malicious or compromised nodes, data tampering, and replay attacks [37,38,39].

Such threats could affect the reliability of the estimated blind-spot geometry. For example, corrupted vehicle widths would alter the estimated ground area and volume, while errors in vehicle positions or yaw could lead to incorrect spatial placement. Under the fixed visibility parameters used in this study, bounding-box errors do not directly change the estimated blind-spot distance.

Future implementations should therefore consider secure communication, sensor and node authentication, data-integrity verification, timestamp validation, and cross-sensor consistency checking to improve the reliability of dynamically updated blind-spot representations.

## 6. Conclusions

This study developed a geometric framework for quantifying and spatially representing vehicle frontal blind spots using ground-truth (GT) 3D bounding boxes from the TUMTraf roadside perception dataset. The framework combines vehicle-specific GT geometry with representative driver-visibility parameters to estimate frontal blind-spot distance, ground area, and volume. Under the adopted fixed visibility parameters, GT vehicle width determines variation in the estimated ground area and volume, whereas bounding-box position, dimensions, and orientation provide the spatial reference needed to place the resulting geometry in the road scene. Calibration-based camera projection was used to examine the spatial consistency of this placement.

The experimental analysis included 214 geometrically valid CAR instances. Replacing vehicle-specific GT widths with the mean-width baseline resulted in a width MAE of 0.108 m, a MAPE of 5.8%, and a maximum relative error of 38.3%. The corresponding mean absolute differences in estimated blind-spot ground area and volume were 0.923 m^2^ and 0.476 m^3^, respectively. Sensitivity analysis showed that increasing the assumed frontal obstruction height from 1.030 to 1.160 m increased the estimated blind-spot distance from 8.583 to 21.091 m. The joint sensitivity analysis further demonstrated that combinations of driver eye height, eye-to-front distance, and frontal obstruction height can substantially affect the absolute estimates, particularly near singular parameter configurations.

The multi-frame, multi-camera projection search identified 172 projectable vehicles among the 214 valid CAR instances. In the retained exploratory validation sample of 27 vehicles, all three assessors classified 24 cases (88.9%) as Valid or Partially Valid. However, agreement on individual four-category verdicts was moderate (Fleiss’ κ=0.512), and the retrospective sampling audit identified inconsistencies between the preliminary sampling pool and the final projectable population. Therefore, the qualitative results should be interpreted as descriptive evidence of spatial placement consistency within the evaluated cases, rather than as population-level estimates or quantitative measurements of actual driver visibility.

Overall, the proposed framework provides a compact geometric representation that connects vehicle-specific GT 3D bounding-box information with simplified frontal blind-spot quantification and spatial placement. Nevertheless, the absolute blind-spot estimates remain conditional on representative visibility assumptions, and camera-based spatial consistency does not establish physical blind-spot accuracy. Future work should incorporate vehicle-specific driver-viewpoint and frontal obstruction measurements, independent direct-visibility ground truth, and estimated 3D bounding boxes to evaluate the effects of perception uncertainty on the resulting blind-spot geometry.

## Figures and Tables

**Table 1:** Comparison of representative vehicle visibility assessment methods and the proposed framework.

Method	Required Inputs	Outputs	Validation
Marshall et al. [13]	Vehicle CAD, digital human model	3D visibility volume	Digital-model evaluation
Summerskill et al. [2]	Heavy-vehicle CAD, driver model	Blind-spot regions	Digital-model evaluation
Drake et al. [17]	Vehicle-specific images	Blind-zone distance	Physical measurement comparison
Mueller et al. [11]	Camera images, eye-position conditions	Direct-visibility maps	Measurement-based comparison
Proposed framework	GT 3D bounding boxes, assumed visibility parameters	Distance, area, volume, spatial placement	Width comparison, sensitivity, camera projection

**Table 2:** Representative visibility parameters used in the geometric model.

Parameter	Definition	Value	Basis
$h_{e}$	Driver eye height above ground	1.27 m	Dupuis et al. [9]
*d*	Longitudinal distance from the ground projection of the driver eye point to the frontal reference point	2.00 m	Capaldo et al. [10]
$h_{f}$	Height of the frontal vision-obstruction point	1.03 m	Representative baseline value adopted in this study

**Table 3:** Overall vehicle and frontal blind-spot characteristics of the final valid CAR sample (n=214) under the baseline parameter setting.

Characteristic	Value
Number of valid CAR instances	214
GT vehicle width, mean (SD)	1.902 (0.170) m
GT vehicle width, min–max	1.376–2.626 m
Frontal blind-spot distance, *D*	8.583 m
Blind-spot ground area, mean (SD)	16.328 (1.459) m^2^
Blind-spot ground area, min–max	11.809–22.538 m^2^
Blind-spot volume, mean (SD)	8.409 (0.751) m^3^
Blind-spot volume, min–max	6.082–11.607 m^3^

**Table 4:** Frontal blind-spot characteristics under the three frontal-obstruction-height scenarios.

Scenario	$h_{f}$ (m)	*D* (m)	Change	Valid/Total	Mean *A* (m^2^)/Mean *V* (m^3^)
Lower $h_{f}$	0.965	6.328	−26.3%	214/215	12.038/5.808
Baseline $h_{f}$	1.030	8.583	–	214/215	16.328/8.409
Higher $h_{f}$	1.160	21.091	+145.7%	214/215	40.122/23.271

**Table 5:** Quantitative effects of replacing vehicle-specific GT widths with the mean-width baseline.

Quantity	Metric	Value
GT vehicle width	Mean width	1.902 m
	MAE	0.108 m
	MAPE	5.8%
	Maximum relative error	38.3%
Blind-spot ground area	Mean absolute difference	0.923 m^2^
	Maximum absolute difference	6.210 m^2^
Blind-spot volume	Mean absolute difference	0.476 m^3^
	Maximum absolute difference	3.198 m^3^

**Table 6:** Qualitative spatial validation outcomes across three assessors for the 27 sampled vehicles.

Validation Outcome	Assessor 1	Assessor 2	Independent Assessor
Valid	12	17	14
Partially Valid	12	7	10
Invalid	0	1	1
Undeterminable	3	2	2
Valid + Partially Valid	24 (88.9%)	24 (88.9%)	24 (88.9%)

## Data Availability

Restrictions apply to the availability of these data. The R2_S1 and R2_S3 sequences of the TUMTraf Intersection Dataset (Release 2) used in this study are third-party data and are available from the TUMTraf Dataset website upon registration. The data can be accessed through the dataset registration page, https://a9-dataset.innovation-mobility.com/en/register (accessed on 23 September 2026) (dataset overview: https://innovation-mobility.com/en/project-providentia/a9-dataset/; accessed on 23 September 2026). Access to and use of the dataset are subject to the terms and conditions specified by the dataset provider. Users of the dataset are requested to cite the associated dataset publication [29].
